# Engineering *Escherichia coli* biofilm to increase contact surface for shikimate and L-malate production

**DOI:** 10.1186/s40643-021-00470-7

**Published:** 2021-11-30

**Authors:** Qiang Ding, Yadi Liu, Guipeng Hu, Liang Guo, Cong Gao, Xiulai Chen, Wei Chen, Jian Chen, Liming Liu

**Affiliations:** 1 State Key Laboratory of Food Science and Technology, Jiangnan University, 1800 Lihu Road, Wuxi, 214122 China; 2 International Joint Laboratory On Food Safety, Jiangnan University, Wuxi, 214122 China

**Keywords:** Biofilm, Contact surface, Self-assembly, Biohybrid, Shikimate, L-malate

## Abstract

Microbial organelles are a promising model to promote cellular functions for the production of high-value chemicals. However, the concentrations of enzymes and nanoparticles are limited by the contact surface in single *Escherichia coli* cells*.* Herein, the definition of contact surface is to improve the amylase and CdS nanoparticles concentration for enhancing the substrate starch and cofactor NADH utilization. In this study, two biofilm-based strategies were developed to improve the contact surface for the production of shikimate and L-malate. First, the contact surface of *E. coli* was improved by amylase self-assembly with a blue light-inducible biofilm-based SpyTag/SpyCatcher system. This system increased the glucose concentration by 20.7% and the starch-based shikimate titer to 50.96 g L^−1^, which showed the highest titer with starch as substrate. Then, the contact surface of *E. coli* was improved using a biofilm-based CdS-biohybrid system by light-driven system, which improved the NADH concentration by 83.3% and increased the NADH-dependent L-malate titer to 45.93 g L^−1^. Thus, the biofilm-based strategies can regulate cellular functions to increase the efficiency of microbial cell factories based on the optogenetics, light-driven, and metabolic engineering.

**Graphical Abstract:**

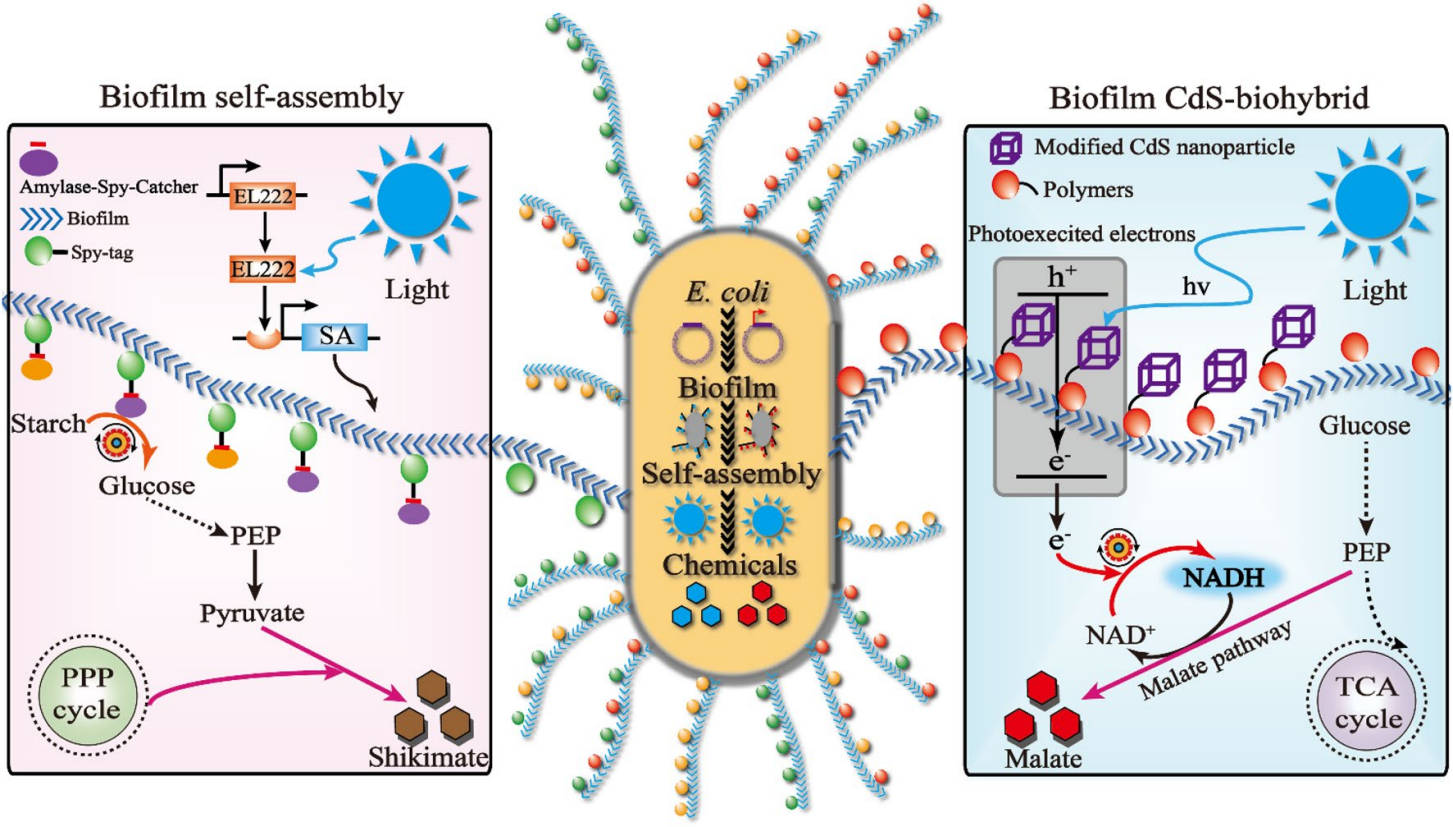

**Supplementary Information:**

The online version contains supplementary material available at 10.1186/s40643-021-00470-7.

## Introduction

Microbial cell factories can utilize reproducible substances in a promising, alternative, and environmentally friendly manner for the production of high-valuable and green chemicals, including food additives, advanced biofuels, and fine pharmaceuticals (Choi et al. 2019; Ko et al. 2020; Lee and Kim 2015; Nielsen and Keasling 2016). To improve the efficiency of microbial cell factories, various strategies at the DNA level (genome engineering and promoter engineering) (Choe et al. 2019; Ohta et al., 1991; Walker et al. 1999), RNA level (RNA ribozyme switch and transcription factor engineering) (Alper and Stephanopoulos 2007; Choi et al. 2019; Pham et al. 2017), protein level (protein engineering and cofactor engineering) (Li et al. 2020; Quijano-Rubio et al. 2021; Titorenko et al. 2002), metabolite level (scaffold engineering) (Niemeyer et al. 1994; Ellis et al. 2019), and cellular level (organelle engineering, morphology engineering, and consortium engineering) (Avalos et al. 2013; Ding et al. 2020; Honjo et al. 2019; Koizumi et al. 1998) have been developed. In particular, organelles could increase the efficiency of microbial cell factories at the cellular level by compartmentalizing key enzymes into targeted sub-organelles to improve enzymatic reaction efficiencies (Hammer and Avalos 2017).

To increase the enzymatic reaction efficiency by using sub-organelles, various strategies have been utilized, including extracellular organelles, in which transport proteins regulate the movement of substances across the cell wall by rewiring functional membrane microdomains or vesicles (Dueber et al. 2009; Sandoval and Papoutsakis 2016; Yang et al. 2021); intracellular organelles, including mitochondria, peroxisomes, and endoplasmic reticulum, to improve intermediate product concentrations and accelerate enzymatic reactions (Avalos et al. 2013) (DeLoache et al. 2016; Grewal et al. 2020); and artificial organelles, such as phase-separated droplets engineered to increase the transformation efficiency by concentrating key pathway enzymes into a compartment (Castellana et al. 2014; Kuska and O'Reilly 2020; Zhao et al. 2019). However, few studies have focused on the contact surface as a physiological parameter of extracellular organelles and a key determinant of the efficiency of microbial cell factories (Nguyen et al. 2014; Sakimoto and Yang 2016; Zhang et al. 2018). Biofilms, as an extracellular organelle, can form on the cell surface to promote the self-assembly of enzymes or nanoparticles for increased substrate utilization (Jiang et al. 2018; Nguyen et al. 2014; Olmez et al. 2019; Wei et al. 2018). Chemical biosynthesis by *Escherichia coli*, *Pseudomonas putida,* or *Bacillus subtilis* could be effectively improved by using biofilms to regulate the contact surface, thereby improving the enzymatic reaction efficiency and chemical production (Benedetti et al. 2016; Leonov et al. 2021; Nguyen et al. 2014).

Biofilms are important microbial organelles that can be regulated by biofilm formation genes, e.g., the curli nanofiber genes, including the biofilm structure gene *csgA*, nucleation gene *csgB,* processing genes *csgE* and *csgF,* secretion genes *csgC* and *csgG,* and direct transcriptional regulatory gene *csgD* to form bacterial biofilms (Kay et al. 2006; Nguyen et al. 2014) and biofilm regulatory genes, including the secondary messenger c-di-GMP, quorum sensing relative signal molecules, and the two component system involved in cellular physiological metabolism (Valentini and Filloux 2016; Yarwood et al. 2004). Moreover, surface biofilms can have multiple functions: improving stress tolerance by forming a protective barrier against changes in the complex environment (Gilbert and Ellis 2019); enhancing biochemical production, which can accelerate bioconversion rates via the immobilized microbial community (Alvarez-Ordóez et al. 2019); improving chemical production via an increased cell density in biofilm-mediated microbial fermentation (Ercan and Demirci 2013); and achieving continuous fermentation by utilizing long-term physical robustness (Cuny et al. 2019; Jiang et al. 2021). These properties are beneficial for the application of biofilms in the microbial biochemical production process. For example, functional peptides (e.g., FLAG, CLP12, and Mms6) fused to the *E. coli* biofilm protein CsgA have been used to construct a biofilm-integrated nanofiber display strategy for improved tolerance and substrate adhesion (Nguyen et al. 2014). *P. putida* biofilms can be activated and degraded via *yedQ* and *yhjH* to degrade haloalkanes (Benedetti et al. 2016). The *B. subtilis* biofilm protein TasA has been assembled into TasA-OPH and TasA-HisTag nanofibers to achieve a two cascade enzyme reaction for transforming PAR to PAP, integrating Ni–NTA-decorated gold nanoparticles (Huang et al. 2019). Therefore, biofilms can be incorporated into self-assembly or biohybrid systems to increase the contract surface and thereby improve enzymatic reaction efficiencies.

In this study, the definition of contact surface is mainly to reinforce the amylase and CdS nanoparticles titer for enhancing the shikimate and L-malate synthesis. Thus, the *E. coli* biofilms were used to form a self-assembly system or CdS-biohybrid system to improve the contact surface for shikimate and L-malate production. First, the optogenetics system was used to control the biofilm-based SpyTag/SpyCatcher system to improve the amylase and glucose concentration for shikimate production. Second, the light-driven system was utilized to regulate the biofilm-based CdS-biohybrid system to improve CdS nanoparticles and NADH concentration for L-malate production. These biofilm-based strategies are promising approaches to increase chemical production by metabolic engineering.

## Materials and methods

### Strains and culture conditions

All plasmids and bacterial strains used in this study are listed in Additional file 1: Tables. Luria–Bertani (LB) broth and Terrific broth (TB) plates were used for choosing a strain. Kanamycin (50 mg L^−1^), ampicillin (100 mg L^−1^), spectinomycin (30 mg L^−1^), and IPTG (500 μM) were added appropriately according to different conditions.

For shikimate production, seed cultures were used for fermentation by transferring fresh colonies to a 50 mL shake flask containing 30 mL LB medium. After culturing for 12 h at 37 °C and 200 rpm, this fermentation solution was inoculated into a 250 mL flask with 50 mL fermentation medium (10 g L^−1^ casamino acids, K_2_HPO_4_ (7.5 g L^−1^), iron ammonium (III) citric acid (0.3 g L^−1^), citric acid monohydrate (2.1 g L^−1^), L-phenylalanine (0.7 g L^−1^), L-tyrosine (0.7 g L^−1^), L-tryptophan (0.35 g L^−1^), and concentrated H_2_SO_4_ (1.2 mL), adding trace elements and amino acids according to this reference (Knop et al. 2001)) with an initial optical density at 600 nm (OD_600_) of 0.1, and then 10 g L^−1^ CaCO_3_ was added as an acid neutralizing agent. For fed-batch cultures in a 3.6 L fermenter, seed cultures were used for fermentation by transferring fresh colonies to a 250 mL flask containing 50 mL LB medium. Shikimate fermentation was carried out in a 3.6 L fermenter containing 1.5 L fermentation medium with 10% inoculum size and 10% w/v starch. Shikimate fermentation was maintained at pH 6.0, rotate rate 650 rpm, air flow 1 vvm, and 37 °C by the automatic addition of 4 M NaOH or 2 M HCl.

For L-malate production, seed cultures were used for fermentation by transferring fresh colonies to a 50 mL shake flask containing 30 mL LB medium (10 g L^−1^ casamino acids). After culturing for 12–18 h at 37 °C and 200 rpm, this fermentation solution was inoculated into a 35 mL flask with 20 mL NBS medium to anaerobic fermentation with OD = 10 (adding 60 g L^−1^ glucose,). And then to 100 g/L NaHCO_3_ maintain the solution pH = 7 and releasing redundant carbon dioxide gas for every 12–24 h, respectively. For fed-batch cultures in a 3.6 L fermenter, seed cultures were used for fermentation by transferring fresh colonies to a 250 mL flask containing 50 mL LB medium. L-malate fermentation was carried out in a 3.6 L fermenter containing 1.5 L fermentation medium and 60 g L^−1^ initial glucose, and then supplied with 800 g L^−1^ glucose at 36 h. L-malate fermentation condition was maintained at pH 6.5, rotate rate 100–200 rpm for anaerobic condition, and 37 °C by the automatic addition of 100 g/L NaHCO_3_.

### DNA manipulation and plasmid construction

Gene deletions were performed according to the Red homologous recombination method. All plasmids were constructed using basic molecular cloning techniques and Gibson assembly. To construct the shikimate biosynthesis pathway, it used the previous strain*.* For constructing the L-malate biosynthesis pathway, the strain is the previous reported. The *el222* gene was constructed by Suzhou Genewiz Biotechnology with codon optimization (Jayaraman et al. 2016, 2018). Primers used in this study are listed in Additional file 1: Tables.

### Light culture condition

The optogenetics illumination for gene expression and fermentation were carried out with blue light LED panel (MODEL: HF-FX160, square light source), which placed 4 cm from cell culture. The light intensity can be regulated by switching power supply with a controller. The applicability of scale fermentation can be improved through utilizing a wrap-round illumination way to increasing the light penetration with two or three blue light sources to surround 3.6 L fermenter.

According to light-driven experiments, the blue light was used to activate the electronics generation for NADH regeneration, which placed 4 cm far from shake flask. The process of biohybrid system assembly can be learned from. *E. coli* cells containing biohybrid CdS or InP nanoparticles were harvested from LB medium by centrifugation (4000 rpm for 10 min). The reaction was initiated by blue light irradiation and stopped by centrifugation and separation of *E. coli*–CdS nanoparticles or *E. coli*–InP nanoparticles (Guo et al., 2018; Jin et al. 2021; Wei et al. 2018).

Total NADH content, NADH content, and NAD^+^ content of fermentation solution were detected and followed by Biyuntian Cofactor Determination Kit.

### Biofilm formation, congo staining, and alcian-congo staining

The cells were then streaked or spotted onto YESCA medium, containing 10 g L^−1^ of casamino acids, 1 g L^−1^ of yeast extract, or seed cultures were inoculated from glycerol stocks and grown in LB Miller medium for 12 h at 37 °C. Experimental cultures were grown at 30 °C and 180 rpm in M63 minimal medium supplemented with 1 mM MgSO4 and with 0.2% w/v glucose or 0.2% w/v glycerol (Huang et al. 2019; Nguyen et al. 2014).

For congo red (CR) staining, the stains were grown in YESCA or M63 minimal medium for 12 h at 30 °C and 180 rpm. Subsequently, 10 μL cultures were spotted onto YESCA- or M63-CR plates, supplemented with 25 μg/mL of CR and 5 μg/mL of Brilliant Blue G250. The plates were then imaged to determine the extent of CR binding after 48 h of incubation at 30 °C. Curli-producing bacteria form red colonies, whereas non-producing cells form white colonies.

For alcian-congo staining, the culture conditions of strains were similar to congo staining. Alcian Blue (AB) Dye: 2 g Alcian blue, 3 mL glacial acetic acid, 97 mL distilled water; (2) Congo red dye: Congo red 2 g, distilled water 100 mL Congo red-alcian blue staining method: a little normal saline was dropped on a clean slide; the bacteria were picked up and mixed them with the inoculation needle. Then, it was added to the same amount of AB dye with normal saline, and it was mixed well and let stand for 3–5 min. Finally, a little congo red dye was added to dye cell, and then the results can be observed from the oil mirror under the fluorescence microscopy.

### BLRS implementation

Blue light repression system (BLRS) was constructed. Additional file 1: Data S1–S3 showed the names and sequences of BLAS. In BLAS, two plasmids, P_Tac_-EL222 and PJ-mKate, were used as input plasmids with various blue light illuminations.

To test BLRS, a colony of BLRS cells containing P_Tac_-EL222 and PJ-mKate was inoculated into medium with the corresponding ampicillin and spectinomycin for overnight (12 ~ 14 h) at 37 °C. Then, seed cultures were inoculated in the refresh LB for 12 ~ 14 h under the different blue light (450 nm) illuminations. This experiment was repeated with a different starting colony for three biological replicates. All cultures were grown in 50 mL medium in 250 mL shake flasks at 200 rpm. Finally, fluorescence density was analyzed (see below).

### Analytical methods

The OD_600_ was measured using a spectrophotometer. Glucose analysis was quantified by the biosensor SBA-90E biological sensor. Shikimate was determined by high-performance liquid chromatography using an Aminex HPX-87H column (7.8 × 300 mm; Bio-Rad Laboratories, Inc., Hercules, CA, USA) at 60 °C with 0.05 mM sulfuric acid as the mobile phase. The injection volume was 20 μL, and the flow rate was 0.6 mL min^−1^. L-malate was determined by high sulfuric acid as the mobile performance liquid chromatography using the same column at 45 °C with 0.05 mmol l^−1^ phase. Bacterial cells were harvested by centrifugation at 8000 r min^−1^ for 10 min. The supernatant was discarded, and then cells were washed twice with 20 mL distilled water.

### ICP-MS and XPS assay

For XPS assay, the specific operation is to grind the sample into fine powder, and it was spread on the aluminum foil. Then, it was covered into a piece of aluminum foil and flattened through hydraulic press. Finally, it was cut into a certain shape with scissors and wait for the test.

For ICP-MS, the samples need to do the digestion, all organic substances are completely digested as far as possible, and the solid and liquid samples are converted into stable and clarified acidic aqueous solution (2% nitric acid v/v), without sol and precipitation. In order to prevent the clogging of the capillary atomizer tube, a microporous filter membrane of 0.22/0.45 um is needed (Wei et al. 2018).

### Flow cytometer assays

For flow cytometer analysis, *E. coli* cells were washed twice with PBS, and then resuspended to an OD_600_ of 0.2. The assays were performed by a LSR Fortessa instrument using PE-TxRed (mKate) and GFP channels. The voltage gains for each detector were set to PE-TxRed, 650 V or FITC, and 407 V. Compensation was performed using cells without mKate or GFP. For each sample, at least 20,000 counts were recorded using a 0.5 mL s^−1^ flow rate. A gate was previously designed based on forward and side scatter (> 99% cells were chosen for the analysis of fluorescence density percentage).

### Assay of qPCR measurements

Measurement of the mRNA level of mKate was based detected by qPCR. Primers were used for amplifying the DNA region proximal to the mKate. The qPCR reactions were performed with a SuperReal Premix SYBR Green Plus kit according to its manual. For each PCR reaction, 15–20 μL sample contained 10–15 ng of DNA, 0.6 μM of each primer, and 10 μL of 2 × SYBR Green Supermix. The reaction process was carried out in an Opticon 2 Real-time PCR system based on the directions: 95 °C for 3 min, followed by 40 cycles of 95 °C for 30 s, 60 °C for 30 s, and 72 °C for 30 s. The qPCR product was checked in a 1.5–2% agarose gel to ensure the efficiency of PCR amplification. The 16S rRNA was used as the reference gene to normalize the expression level of targeted gene. For each RNA preparation, at least three independent real-time PCR measurements were performed, respectively. RT-PCR primers are listed in Additional file 1: Tables.

### The bioimage of agarose plates

*E. coli* strains, including BLRS systems, were cultured overnight, respectively. A photomask was placed on the bottom side of the prepared agar plate and used aluminum oxide to avoid blue light illumination. Seed cultures were transferred to fresh medium for OD_600_ = 0.5–0.8, and then 400–600 μL bacteria cultures were taken to plate for bioimaging assay. The whole setup was kept inside the incubator at 37 °C. The LB plates were illuminated under 450 nm blue light or dark condition for 36–48 h.

### Cell viability assays

The viability of cells in LB plates were assessed by inoculating cells in fermentation medium. Cells were diluted to OD_600_ = 0.5 and 10 μL of 10 × serially diluted cell suspension was spread on each agar plate with different strains. Then, the LB plates were cultured with 37 °C for 12 h. Next, the numbers of living cell in different culture times and different strains were calculated, respectively.

### Enzymatic assays

As for amylase assay, after 12 h of growth, each sample was collected from cultures via centrifugation at 4 °C for 15 min, and the 200 μL enzyme solution was taken into solution. Enzymatic activity of α-amylase was quantitatively determined based on the 3,5-dinitrosalicylic acid method by using 200 μL reaction mixture containing 0.5 wt% soluble starch in 20 mM sodium acetate buffer and 50 μL enzyme solution at pH = 5.5. The pH of the reaction mixture was controlled by using 5 M NaOH solution. Enzymatic reactions were terminated by adding a solution consisting of 0.4 M NaOH, 22 mM 3,5-dinitrosalicylic acid, and 1.1 M potassium sodium ( +)-tartrate tetrahydrate incubated for 5 min. And then the DNS method was used to detect the enzyme activity.

### Assay of congo red degradation

InP and CdS nanoparticles were purchased from Aladdin web. 60 mg/L congo red solution was cultured with *E. coli* and nanoparticles during the 450 nm blue light culture. After culturing 90 min, the sample was collected to detect the congo red degradation in the 540 nm by using a SpectraMax M3 plate reader.

### Assay of fluorescence intensity

The engineered *E*. *coli* strains used for assaying fluorescence intensity were plated on the LB plates for overnight at 37 °C, 200 rpm. After that, they were inoculated into 50 mL fresh LB with 1–2% inoculum size (vol/vol) and then cultured at 37 °C, 200 rpm for 12–16 h. For assaying fluorescence intensity, the fluorescence of cell culture was detected by a SpectraMax M3 plate reader. The excitation and emission wavelengths of mKate gene was set at 588 ± 10 nm and 645 ± 10 nm, respectively.

## Results

### Screening of *E. coli* biofilm genes

**Fig. 1:**
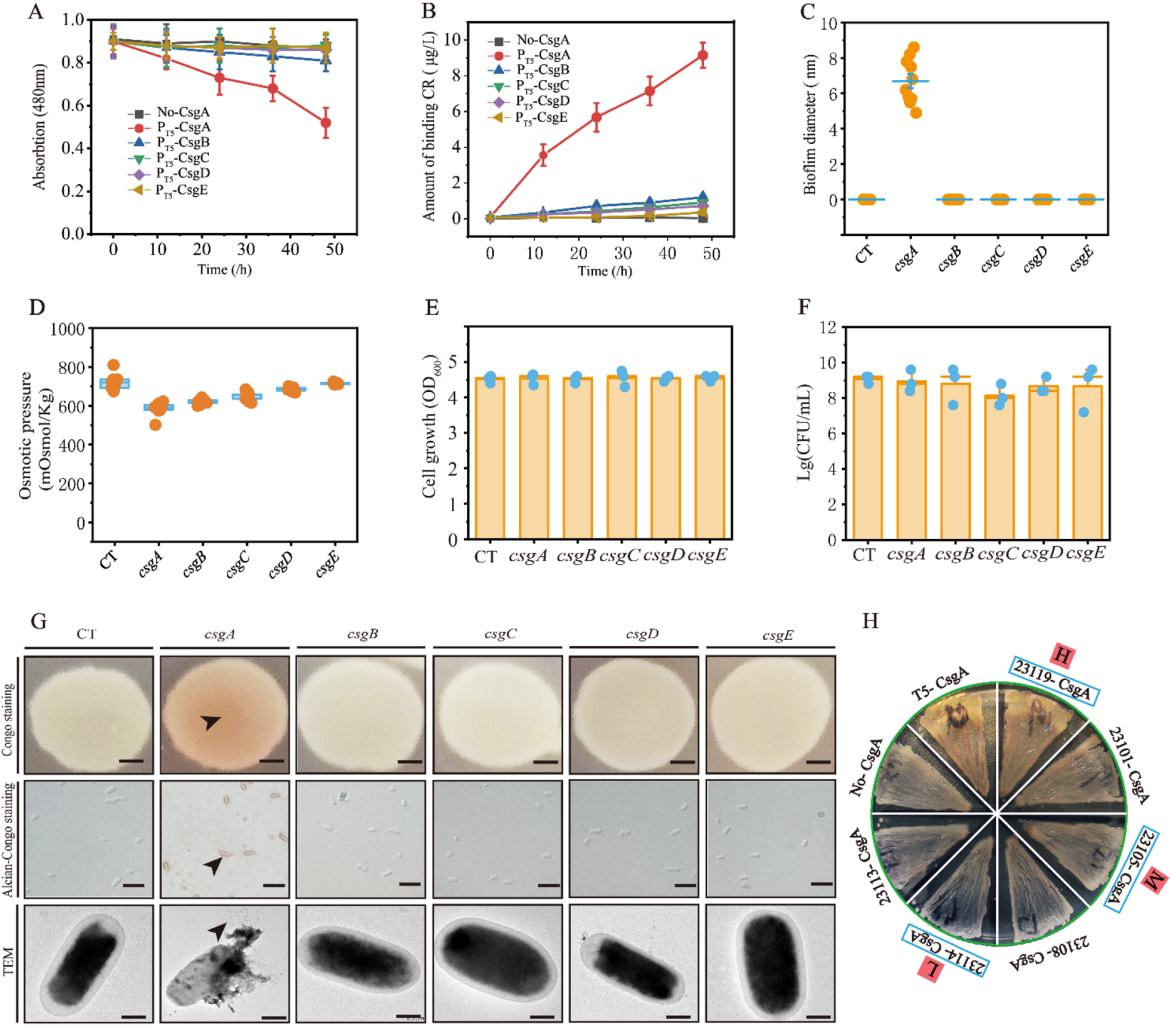
Characterization of *E. coli* biofilm synthesis by genetic manipulation. **A** The absorption of congo red with different biofilm genes in *E. coli* MG1655. **B** Comparing the amount of binding congo red with different biofilm genes in *E. coli* 1655. **C** Comparing the biofilm diameter with different biofilm genes in *E. coli* MG1655.** D** The osmotic pressure with different biofilm genes in *E. coli* MG1655. **E** The changes of CFU with different biofilm genes in *E. coli* MG1655. **F** The changes of cell growth with different biofilm genes in *E. coli* 1655.** G** Comparing the congo red staining, alcian-congo staining, and TEM with different biofilm genes in *E. coli* MG1655.** H** Comparing the different promoters to control biofilm *csgA* expression in *E. coli* 1655, “H” is high strength, “M” is medium strength, “L” is low strength. For A–D, *n* = 3. Error bars, mean ± s.d. Scale bar for congo staining is 1 cm, Scale bar for congo staining is 2 mm, Scale bar for Alcian-Congo staining is 5 μm, Scale bar for TEM is 1 μm

To obtain an available and controllable biofilm system, the five genes involved in *E. coli* biofilm formation were screened to obtain the biofilm. As illustrated in Fig. 1, *csgA*, *csgB*, *csgC*, *csgD,* and *csgE* were each overexpressed in *E. coli* MG1655 to induce biofilm formation. An obvious difference was observed in the engineered strains overexpressing *csgA*. The standard amyloid-staining colorimetric dye Congo red was used to determine the extent of biofilm production through strongly binding the curli nanofiber (Jiang et al. 2018; Nguyen et al. 2014). Thus, the absorption peak of Congo red and osmotic pressure was 43.47% and 20.46% lower in the engineered strains overexpressing *csgA* than in the control strain, respectively (Fig. 1A, D). Furthermore, the amount of bound Congo red and biofilm diameter for the engineered strain overexpressing *csgA* increased to 9.21 ng and 6.7 nm, respectively (Fig. 1B, C). However, the cell growth (OD_600_) and colony-forming unit (CFU) of *E. coli* was not changed significantly (Fig. 1E, F). The biofilm was represented and identified by Congo red staining, alcian-congo red staining, and transmission electricity microscopy (TEM) (Fig. 1G).

To confirm the controllability of *E. coli* biofilm formation, *csgA* was controlled by promoters with different strengths, resulting in differences in the amount of bound Congo red. Thus, this demonstrates that the positive relations between the amount of bound Congo red with stronger biofilm expression (Fig. 1H). These results demonstrate that *csgA* is an efficient and controllable target to increase the contact surface for Congo red absorption.

### Increasing the contact surface by biofilm self-assembly and CdS-biohybrid systems

After validating the effect of the *E. coli* biofilm gene *csgA*, two distinct groups were established. In the first group, the biofilm gene was overexpressed and a split-adhesion system was used, in which a 13-amino-acid peptide (SpyTag) forms a highly specific and spontaneous isopeptide bond with a 15 kDa protein (SpyCatcher) to construct a biofilm-based self-assembly system. In the second group, the surface of the *E. coli* biofilm and CdS nanoparticles was modified by polyphenol functionalization, resulting in programmed adhesion to CdS nanoparticles to construct a CdS-biohybrid system.

**Fig. 2:**
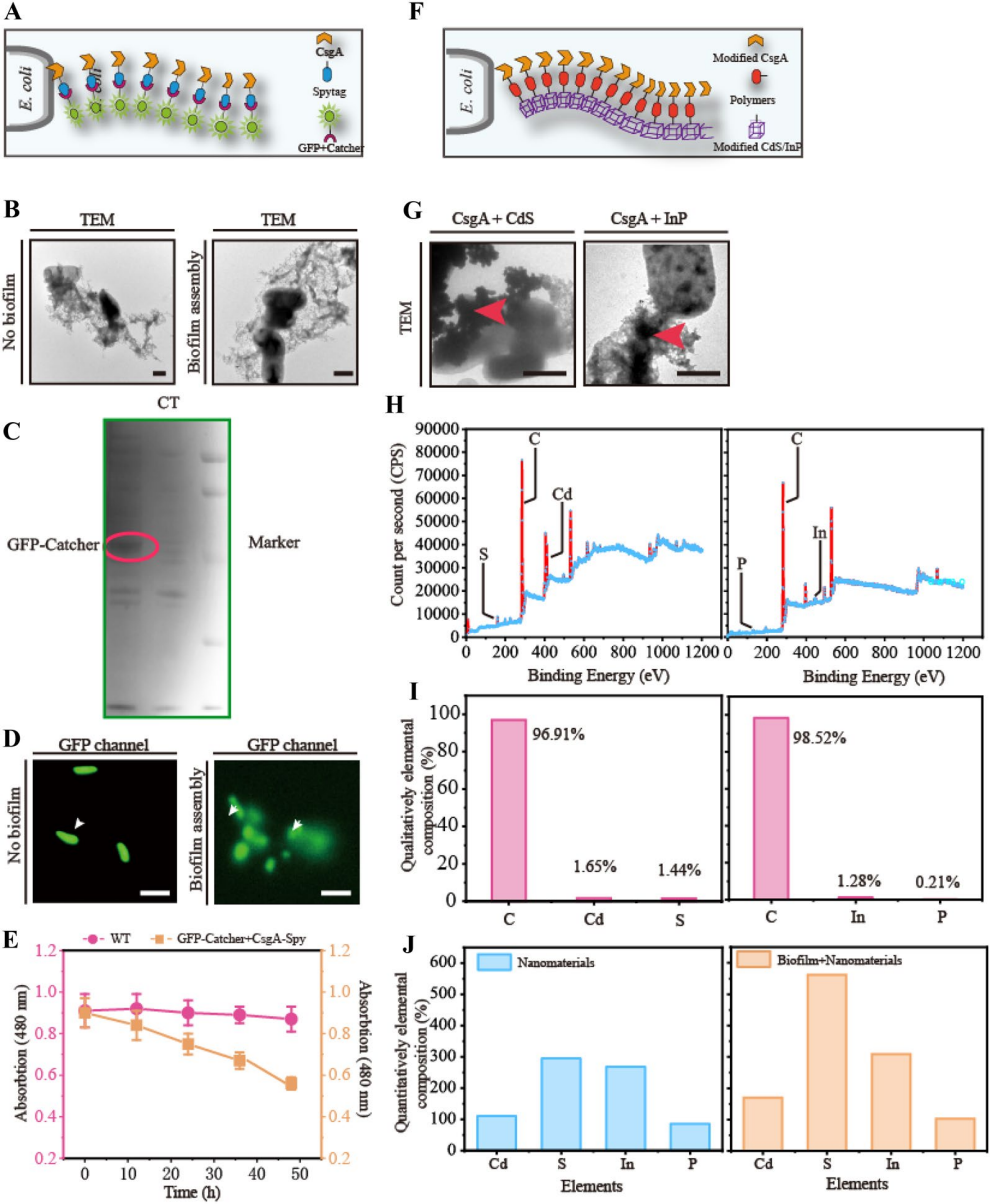
Self-assembly of covalent immobilization proteins and nanoparticles based on biofilms. **A** The schematic diagram of self-assembly of Spy-Tag with GFP-catcher in *E. coli* MG1655. **B** The TEM exhibition in different *E. coli* MG1655.** C** The SDS-page exhibition in different *E. coli* MG1655. **D** Comparing the changes of assembly and no-assembly biofilm, expression and no-expression system by fluorescence detection. **E** The changes of absorption with or without biofilm.** F** The schematic diagram of self-assembly of nanoparticles with *E. coli*.** G** Comparing the TEM with InP or CdS in *E. coli* MG1655 biofilm. **H**, **I** The confirmation and atom account of randomly chosen biohybrids through XPS analysis. **J** Detecting concentration of Cd, S, In, and P in different *E. coli* through ICP-MS. For C, *n* = 3. Error bars, mean ± s.d. Scale bar for **B**, **G** is 1 μm, scale bar for (**D**) is 5 μm,

A biofilm-based SpyTag/SpyCatcher self-assembly system was developed to improve the contact surface for heterogeneous proteins (Fig. 2A). To demonstrate that the SpyTag/SpyCatcher system can assemble with the biofilm on the cell surface, the CsgA-SpyTag fused protein and GFP-SpyCatcher fused protein were overexpressed and secreted to form the biofilm self-assembly system for GFP protein expression on the cell surface. The following results were obtained: (i) SDS–PAGE and TEM demonstrated the expression and extracellular secretion of this biofilm self-assembly system (Fig. 2B, C); (ii) fluorescence microscopy observations showed GFP expression on the *E. coli* surface (Fig. 2D); and (iii) the absorbance of Congo red decreased to 0.54 after biofilm self-assembly (Fig. 2E). Based on these results, the biofilm-based SpyTag/SpyCatcher protein self-assembly system can improve the contact surface by fixing the heterogeneous proteins on the extracellular biofilm.

Another biofilm-based CdS-biohybrid system was constructed to increase the contact surface for CdS nanoparticles (Fig. 2F and Additional file 1: Figure S1). Two typical light-harvesting nanoparticles, InP and CdS, and *E. coli* biofilm CsgA were modified using the polyphenol-based method (Guo et al., 2018). The nanoparticles were functionalized to attach to *E. coli* CsgA for display on the cell surface (Fig. 2G and Additional file 1: Figure S1). We obtained the following key results. (i) The CdS and InP nanoparticles were efficiently attached to the extracellular biofilm, as determined using TEM (Fig. 2G). (ii) The qualitative elemental composition of CdS and InP nanoparticles in the biofilm-based biohybrid system levels were 1.44, 1.65, 1.28, and 0.21% of S, Cd, In, and P, respectively, as determined using XPS (Fig. 2H, I). (iii) A quantitative elemental composition analysis of CdS and InP nanoparticles in the biofilm-based biohybrid system showed improvements to 53.64, 112.07, 15.58, and 19.26% of Cd, S, In, and P, respectively, using ICP-MS, demonstrating that the CdS nanoparticles exhibited a high biofilm attachment capacity (Fig. 2J). Based on the above results, the CdS nanoparticle was effective for forming the biofilm-based CdS-biohybrid system. These results confirmed that the biofilm-based CdS-biohybrid system can improve the contact surface through reinforce the CdS nanoparticle titer on the biofilm.

### Reinforcing shikimate production via increased amylase and glucose concentrations with an improved contact surface

**Fig. 3:**
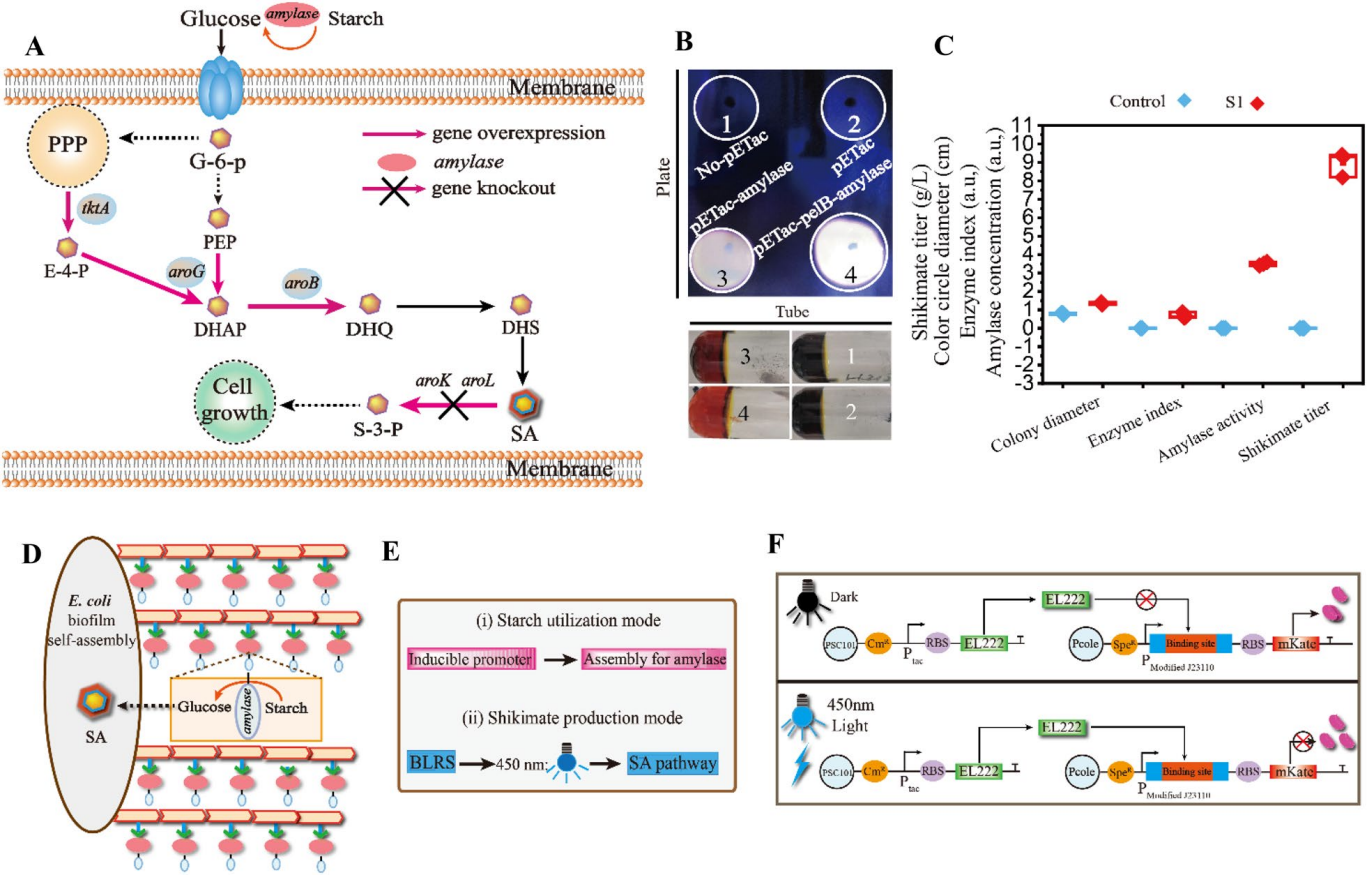
Engineering shikimate synthesis by biofilm self-assembly. **A** The schematic diagram of shikimate biosynthesis pathway in *E. coli* S1. **B** Comparing the color circle with starch in different *E. coli*. **C** The change**s** of color circle diameter, enzyme index, amylase concentration, and shikimate titer in different *E. coli*. **D** Conceptual graph of starch utilization with biofilm-based SpyTag/SpyCatcher covalently protein system for L-malate production.** E** Conceptual graph of two modes fermentation for shikimate production.** F** The schematic diagram of BLRS, containing the plasmid Ptac-EL222 and PJ-mKate. For C, *n* = 3. Error bars, mean ± s.d. Each gene encodes the following: Glu: glucose; G6P: glucose-6-phosphate; PYR: pyruvate; PEP: phosphoenolpyruvate; AcCoA: acetyl-CoA; E4P, erythrose-4-phosphate; DAHP, 3-deoxy-D-arabino-heptulosonate-7-phosphate; Ru5P, Ribulose 5-phosphate; DHQ, 3-dehydroquinic acid; DHS, 3-dehydroshikimate; SA, shikimate

An engineered *E. coli* S1 was constructed, in which the *aroB, aroG,* and *tktA* genes involved in the shikimate synthesis pathway were overexpressed and amylase concentration was increased (Fig. 3A). Then, starch utilization by the engineered *E. coli* S1 was analyzed. (1) The color ring of *E. coli* S1 was 68.35% larger than that of the wild type (Fig. 3B). (2) The enzyme index, amylase concentration, and glucose concentration of *E. coli* S1 were 0.7, 3.5 U, and 45 g L^−1^, respectively (Fig. 3C, Additional file 1: Figure S2). (3) Shikimate titer and productivity of *E. coli* S1 were 10.24 g L^−1^ and 0.14 g L^−1^ h^−1^, respectively (Fig. 3C). These results showed that a low glucose concentration may limit shikimate production, and this was associated with a low amylase concentration. To resolve the low glucose concentration during shikimate fermentation, the process of shikimate biosynthesis was divided into two modes: (i) starch utilization, which was increased by the self-assembling biofilm with inducible promoters (Fig. 3D) and (ii) shikimate production, in which pathway enzymes were induced by the blue light-regulated expression system (Fig. 3E).

**Fig. 4:**
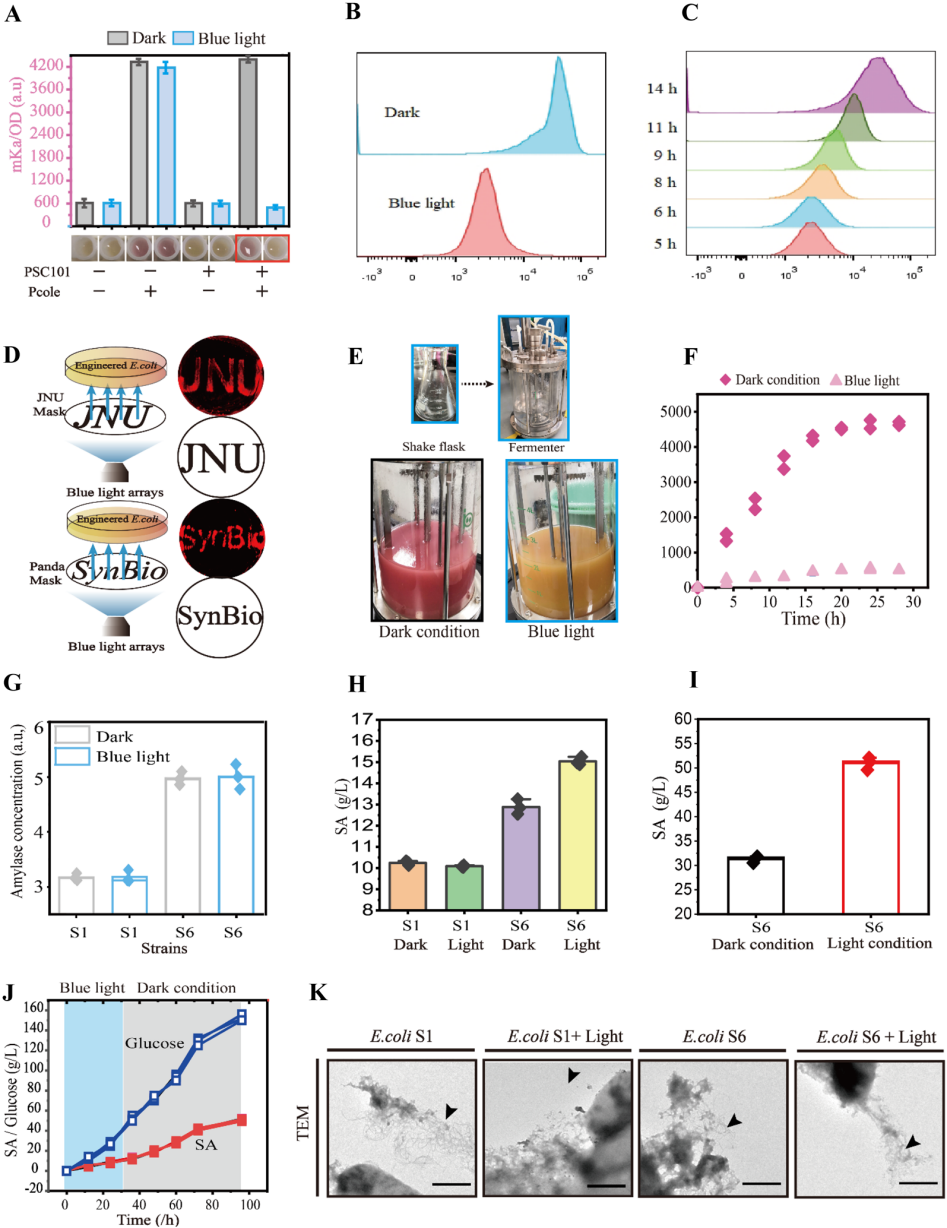
Increasing shikimate production by biofilm self-assembly. **A** Fluorescence repression with BLRS under different conditions. **B**, **C** Fluorescence repression detection through Flow cytometer analysis under the different conditions. **D** Bioimage for “JNU” and “SynBio” was achieved through utilizing BLRS system.** E** The BLRS system scaled up to 5 L fermenter under the blue light and dark conditions.** F** The Fluorescence repression of BLRS system in the 5 L fermenter under the blue light and dark conditions. **G** Comparing the enzyme concentration with different strains. **H** Comparing the shikimate titer with different strains. **I S**hikimate production with 3.6 L fermenter by light stimulation during fed-batch fermentation with *E. coli* S6. **J S**hikimate production in the 3.6 L fermenter with two stage fermentation.** K** The formation of biofilm-based SpyTag/SpyCatcher system of *E. coli* S1 and S6 under the light or dark conditions. For A, G, H, I, *n* = 3. For F, *n* = 2. Error bars, mean ± s.d. Scale bar for (**K**) is 1 μm,

To spatiotemporally control these two modes, a blue light repression system (BLRS) was constructed. As described in previous studies (Jayaraman et al. 2016, 2018), the EL222 light-sensitive protein was controlled by the Ptac promoter, and the central position of the − 35 to − 10 region of the J23110 promoter was replaced with an EL222 binding site, which was then assembled into low copy P_SC101_ and P_cole_ to reduce the metabolic burden (Fig. 3F). Degradation tags of three strengths were used to regulate EL222 expression, and the DAS tag showed the best switch performance (Additional file 1: Figure S3). First, the abundance of mKate was 9.1-fold lower in response to blue light than in dark conditions and showed the homogeneity of this system based on flow cytometer (Fig. 4A–C). Next, SDS–PAGE and RT–PCR were performed to evaluate changes in mKate expression at the protein and transcript levels (Additional file 1: Figures S4 and S5). Then, bioimaging for “JNU” and “SynBio” was performed to display the spatial specificity of BLRS (Fig. 4D). In addition, the different strains, mediums, and light sources were used to show the university and specificity of BLRS (Additional file 1: Figures S5–S7). Furthermore, the optogenetics tool can efficiently regulate heterogeneous and endogenous bacterial genes; the galactosidase, the cell division proteins SulA and FtsZA, and cell lysis gene were chosen to exhibit the changes of the galactosidase activity, cell length, and cell lysis in different blue light conditions (Additional file 1: Figures S8–S10). Finally, the BLRS system was scaled up to 5 L fermenter to show the applicability of optogenetics (Fig. 4E, F). Together, the results demonstrated that BLRS is effective for the regulation of gene expression.

Then, the biofilm-based SpyTag/SpyCatcher system was introduced into *E. coli* S1 to increase the contact surface, leading to improvements in the amylase and glucose concentrations for shikimate production via BLRS regulation*.* This biofilm self-assembly system was controlled by the Ptrc and PT5 promoters. The two modes were established by manipulating the timing of blue light illumination and inducer, respectively (Fig. 4E). This led to the formation of *E. coli* S6. As a result, the amylase and glucose concentrations in *E. coli* S6 under light conditions were 48.57% and 20.07% greater than those of *E. coli* S6 under dark conditions, respectively (Fig. 4G, Additional file 1: Figure S1). This led to increases in the starch-based shikimate titer and *E. coli* S6 productivity under light conditions to 15.04 g L^−1^ and 0.21 g L^−1^ h^−1^, which were 46.88% and 46.88% greater than those of *E. coli* S6 under dark conditions, respectively (Fig. 4H). The culture was scaled up to a 3.6 L fermenter, and the starch-based shikimate titer and *E. coli* S6 productivity under light conditions increased to 50.96 g L^−1^ and 0.71 g L^−1^ h^−1^, which were 62.81% and 62.81% greater than those of *E. coli* S6 under dark conditions (Fig. 4I, J). Finally, the formation of biofilm-based SpyTag/SpyCatcher system of *E. coli* S1 and S6 under the light or dark condition were identified through TEM analysis (Fig. 4K).

### Enhancing L-malate production via increased CdS and NADH concentrations with an improved contact surface

**Fig. 5:**
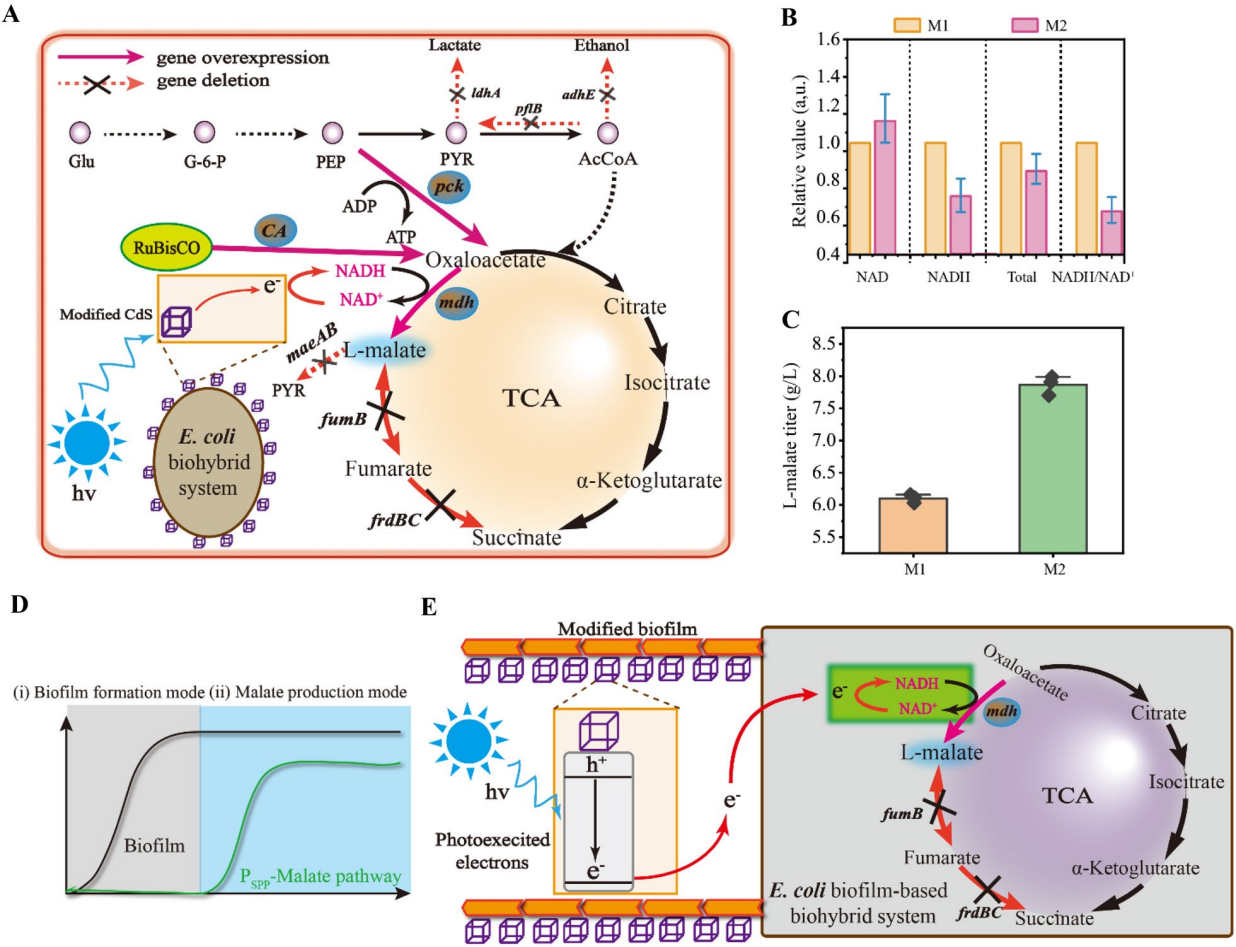
Engineering L-malate biosynthesis by biofilm biohybrid system. **A** The schematic diagram of L-malate biosynthesis pathway in *E. coli* M1. **B** L-malate titer different strains with biofilm biohybrid system. **C** Biohybrids system for transporting electricity for NADH regeneration under blue light illumination. **D** Conceptual graph of two modes fermentation for shikimate production.** E** Conceptual graph of light-driven biofilm-based biohybrid system with NADH regeneration. For B, C, *n* = 3. Error bars, mean ± s.d. Each genes: PCK, phosphoenolpyruvate carboxykinase; MDH, L-malate dehydrogenase

**Fig. 6:**
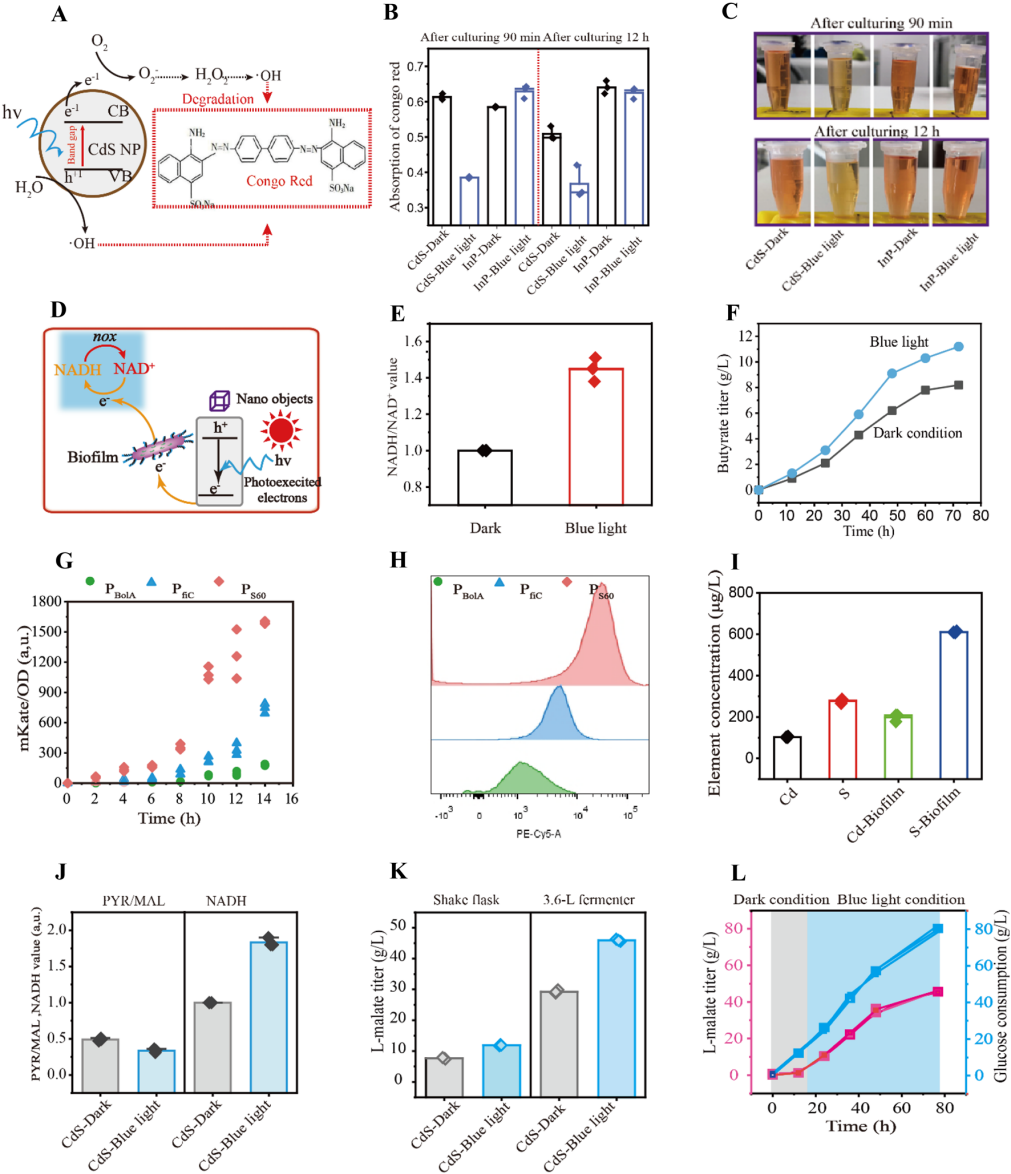
Enhancing L-malate production by biofilm biohybrid system. **A**–**C** The congo red degradation assay with or without blue light irradiation in the CdS- or InP-biohybrid system. **D**, **E** NADH regeneration in NOX overexpressing strain under the dark or blue light conditions. **F** Butyrate titer with CdS-biohybrid system under the blue light or dark conditions. **G** Fluorescence activation with different stationary phase promoters. **H** Fluorescence activation with different stationary phase promoters through flow cytometer analysis.** I** Comparing the CdS nanoparticle concentration with different conditions.** J** Comparing the pyruvate/L-malate and NADH value with different conditions. **K** L-malate production with the engineered *E. coli* M6 controlled by light irradiation during 3.6 L fed-batch fermentation.** L** L-malate production in the 3.6 L fermenter with two-stage fermentation. For B, E-L, *n* = 3

An engineered *E. coli* M1 was constructed in which the *ca*, *pck,* and *mdh* genes involved in the L-malate synthesis pathway were overexpressed, while the key pathway enzyme MDH is a NADH-dependent enzymes (Hu et al. 2018)(Fig. 5A). Thus, the newly constructed CdS-biohybrid system was used for NADH regeneration by light irradiation, leading to *E. coli* M2 formation (Fig. 5A and Additional file 1: Figure S2). Then, NADH metabolism was analyzed in engineered *E. coli* M1 and M2. (1) The NADH concentration and NADH/NAD^+^ ratio of *E. coli* M2 were decreased by 32% and 39% compared to those of *E. coli* M1 (Fig. 5B). (2) The concentrations of Cd and S elements in *E. coli* M2 were only 110.2 μg L^−1^ and 295.3 μg L^−1^ in the biofilm-based biohybrid system (Fig. 2J). (3) The L-malate titer and *E. coli* M2 productivity increased to 7.77 g L^−1^ and 0.11 g L^−1^ h^−1^, showing 27.38% and 27.38% increases compared to those of *E. coli* M1, respectively (Fig. 5C). The decreased NADH concentration may limit L-malate production owing to a low concentration of CdS nanoparticles. To improve NADH regeneration during the L-malate fermentation process, a strategy was introduced to increase electron transfer by dividing the process of L-malate biosynthesis into two modes: (i) a biofilm formation mode, in which biofilm formation was increased by the constitutive expression of *csgA*, and (ii) a L-malate production mode, in which pathway enzymes were expressed by stationary phase promoters and the biofilm-based CdS-biohybrid system under light irradiation (Fig. 6D, E).

To efficiently control these two modes, light-driven system was used to regulate the biofilm-based CdS-biohybrid system for NADH regeneration. Congo red can degraded with electron–hole pairs of biofilm-based CdS-biohybrid system, due to the hydroxyl radicals are generated by the flow of electrons (Jin et al. 2021). Thus, electron transfer in the biofilm-based CdS-biohybrid system under light irradiation was confirmed using a Congo red degradation assay, showing that Congo red was degraded by 37.26% after the assembly of CdS nanoparticles (Fig. 6A–C). The biofilm-based CdS-biohybrid system was introduced into *E. coli* M3 to express NADH oxidase. Thus, the NADH concentration of *E. coli* M3 was 45.6% higher than that of the strain without the biofilm-based CdS-biohybrid system (Fig. 6D, E). Finally, the CdS-biohybrid system was introduced into NADH-dependent butyrate-producing strain, and the result showed that the butyrate titer and NADH value increased by 43% and 52%, compared to that of dark condition (Fig. 6F and Additional file 1: Figure S11). Taken together, these results demonstrated that this biofilm-based CdS-biohybrid system can efficiently regenerate NADH for biomanufacturing.

The biofilm-based CdS-biohybrid system was introduced into *E. coli* M1 to increase the contact surface, resulting in the improvement of CdS and NADH concentrations for L-malate production under light irradiation*.* Different strengths of stationary phase promoters were analyzed via enzyme labeling and flow cytometer to control the expression of L-malate pathway enzymes (Fig. 6G, H). The two modes were established by manipulating the timing of light illumination and stationary phase promoters, respectively. Thus, different stationary phase promoters were combined with the biofilm-based CdS-biohybrid system to obtain *E. coli* M4, *E. coli* M5, and *E. coli* M6. The CdS and NADH concentrations of *E. coli* M6 under light conditions increased by 90.28% (Cd), 120.27% (S), and 83.3%, while the pyruvate/L-malate of *E. coli* M6 was 31% lower than that of *E. coli* M6 under dark conditions (Fig. 6I, J). This led to increases in the L-malate titer and *E. coli* M6 productivity under light conditions to 11.9 g L^−1^ and 0.16 g L^−1^ h^−1^, which were 55.96% and 55.96% higher than those of *E. coli* M6 under dark conditions (Fig. 6K). The culture was scaled up to a 3.6 L fermenter, and the L-malate titer and productivity of *E. coli* M6 under light conditions increased to 45.93 g L^−1^ and 0.59 g L^−1^ h^−1^, which were 56.76% and 56.76% greater than those of *E. coli* M6 under dark conditions (Fig. 6L).

## Discussion

In this study, a biofilm-based SpyTag/SpyCatcher system and a CdS-biohybrid system were developed to improve the contact surface of *E. coli* strains for the production of starch-based shikimate and NADH-dependent L-malate, which integrate the optogenetics system, light-driven, and metabolic pathway. To this end, three strategies were adopted: (i) induction of the expression of the curli nanofiber gene *csgA,* which promotes *E. coli* biofilm formation and possesses multiple functions; (ii) the development of a biofilm-based SpyTag/SpyCatcher protein system and biofilm-based CdS-biohybrid system to improve the contact surface; and (iii) the improvement of the contact surface to increase the amylase and CdS concentrations, thereby increasing the glucose and NADH concentrations and promoting starch-based shikimate and NADH-dependent L-malate production. Upon the implementation of these strategies, the titers of starch-based shikimate and NADH-dependent L-malate increased to 50.96 g L^−1^ and 45.93 g L^−1^, respectively.

In this article, through Congo red absorbance, fluorescence microscopy, and TEM, we observed that the contact surface was improved by the biofilm-based SpyTag/SpyCatcher protein system in which GFP was displayed around the *E. coli* surface or by the biofilm-based CdS-biohybrid system in which CdS nanoparticles were attached to the *E. coli* surface. The restricted contact surface limits the biomanufacturing process; therefore, the bioconversion efficiency can be improved by increasing the contact surface. Previous studies aimed at improving the contact surface have utilized various strategies. For example, glycosylphosphatidylinositol (GPI)-anchored cell wall proteins can attach to heterologous proteins on the cell surface to increase the contact surface between the enzyme and substrate (Inokuma et al. 2020). The cellulosome has been utilized to form a multi-enzyme complex structure on the cell wall to increase lignocellulose degradation (Tsai et al. 2010). Outer membrane proteins have been developed to display specific proteins on the cell surface to increase the contact surface with modified nanoparticles (Wei et al. 2018). The key metabolic pathway has been compartmentalized into natural or artificial organelles for improving the contact surface of enzymes and substrates to enhance the transformation of intermediates to targeted chemicals (Avalos et al. 2013; Reifenrath et al. 2020).

Finally, upon the improvement of the contact surface, the amylase and glucose concentrations increased by 48.57% and 20.07%, leading to 62.81% and 62.81% improvements in the shikimate titer and productivity. The CdS and NADH concentrations were improved by 53.64% (S), 112.07% (Cd), and 83.3%, resulting in 56.76% and 56.76% increases in the L-malate titer and productivity. On one hand, the low amylase concentration limits shikimate production based on starch as a substrate. Previous attempts to improve shikimate production have mainly focused on the following: knocking out *pgi* of *E. coli* in KPM1 SA1/pKPM-SA1 to allow a greater NADPH for key enzymes in the shikimate synthesis pathway (Ahn et al. 2011); inactivating the phosphotransferase system gene ptsHIcrr, shikimate kinases I and II *aroK* and *aroL*, and pyruvate kinase I *pykF* to inhibit downstream gene expression and enhance the upstream carbon flux (Alberto 2011); overexpressing PTS and an endogenous myo-inositol transporter IolT1 and glucokinases to increase the key precursor for shikimate production (Kogure et al. 2016); and utilizing a glucose–xylose co-substrate to decouple cell growth and shikimate synthesis (Fujiwara et al. 2020). On the other hand, NADH regeneration limits L-malate production. Previous studies to increase L-malate biosynthesis have mainly focused on the following: designing pyruvate carboxylation, oxaloacetate reduction, and L-malate transport systems in *Saccharomyces cerevisiae* to increase L-malate production (Zelle et al. 2008); using adaptive laboratory evolution and medium optimization in *Ustilago trichophora* TZ1 to achieve high cell density fermentation for L-malate biosynthesis (Zambanini et al. 2016); engineering *S. cerevisiae* for the transport of dicarboxylic acids by the SpMae1(p) transporter to increase L-malate production (Darbani et al. 2019); and displaying surface CdS nanoparticles on the *E. coli* cell membrane to absorb light energy for NADH regeneration in the NADH-dependent L-malate biosynthesis process (Hu et al. 2021b).

According to study of CdS-biohybrid system: (i) A surface-display biohybrid was constructed through CdS nanoparticle for improving the H_2_ production to 0.52 ± 0.01 mmol/10^8^ cells (Wei et al. 2018). Similarly, the CdS nanoparticle for NADH regeneration with CO_2_ fix (Hu et al. 2021a). (ii) The CdS was synthesized through addition of Cd^2+^ and cysteine was used to attach the electrotroph *M. thermoacetica* for acetate production (Sakimoto and Yang 2016). (iii) The CdS nanoparticle was used to attach the acetogenic *Clostridium autoethanogenum* for improving the NADH/NAD^+^ ratio (Jin et al. 2021). (iv) Cd(NO_3_)_2_ was used to form the CdS nanoparticle to attach the *Clostridium beijerinckii* for producing 10.24 g/L butanol (Wang et al. 2021). As for using CdS-biohybrid system to improve the NADH: (i) Compared to gene expression for NADH regeneration, the CdS-biohybrid system was constructed to generate the NADH not only can decouple the L-malate production and NADH regeneration of endogenous metabolisms, but also can improve the L-malate production in a carbon and energy-efficient utilization way (Jin et al. 2021; Wei et al., 2018). In addition, it was easily to obtain and manipulate the CdS-biohybrid system (Guo et al., 2018; Sakimoto and Yang, 2016). (ii) In this study, the CdS nanoparticle was attached to *E. coli* biofilm through polyphenol-based method. Therefore, the Cd element and S element of CdS-biohybrid system were efficiently increased by 90.28% and 120.27%, which was better than the natural absorption system. (iii) The NADH concentration of this CdS-biohybrid system was increased to 32 μmol/gDCW, which showed an 83.3% improvement, compared to that of control. Furthermore, the L-malate titer and productivity of this CdS-biohybrid system were efficiently increased to 45.93 g/L and 0.59 g/L/h with a lower metabolic burden, which were improved by 56.76% and 56.76%.

Taken together, the biofilm-based regulation strategy provides a platform for improving the contact surface in a controlled spatiotemporal and reversible manner. The biofilm-based SpyTag/SpyCatcher protein system and biofilm-based CdS-biohybrid system not only improve the contact surface but also increase the glucose and NADH concentrations to increase targeted chemical production. This biofilm-based strategy is an attractive approach for the construction of microbial cell factories for high-value chemical production.

### Supplementary Information

**Additional file 1.** Gene sequences used for plasmids and strains construction; **Figures S1–S11** and **Tables S1–S5** were set in the supporting information.

## Data Availability

All data generated or analyzed during this study are included in this article.
