# Supplementary material for: Engineering Escherichia coli biofilm to increase contact surface for shikimate and L-malate production
Source: Bioresour Bioprocess. 2021 Nov 30;8(1):118. doi: 10.1186/s40643-021-00470-7 (PMC10992329; doi:10.1186/s40643-021-00470-7)
Supplement: Supplementary file 1 — Additional file 1. Gene sequences used for plasmids and strains construction; Figures S1–S11 and Tables S1–S5 were set in the supporting information. [file 40643_2021_470_MOESM1_ESM.docx]

### Engineering *Escherichia coli* biofilm to increase contact surface for shikimate and L-malate production

Qiang Ding^1,2^, Yadi Liu^1,2^, Guipeng Hu^1,2^, Liang Guo^1,2^, Cong Gao^1,2^, Xiulai Chen^1,2^, Wei Chen^1,2^，Jian Chen^1,2^，Liming Liu^1,2*^

^1^State Key Laboratory of Food Science and Technology, Jiangnan University, Wuxi 214122, China

^2^International Joint Laboratory on Food Safety, Jiangnan University, Wuxi 214122, China

Corresponding author: Liming Liu

Mailing address: State Key Laboratory of Food Science and Technology, Jiangnan University, 1800 Lihu Road, Wuxi 214122, China

Fax/Tel.: +86-510-85197875.

*E-mail*: [mingll@jiangnan.edu.cn](mailto:mingll@jiangnan.edu.cn)

**This file includes:**

Supplementary Figure 1 to Supplementary Figure 11

Supplementary Table 1 to Table 5

**
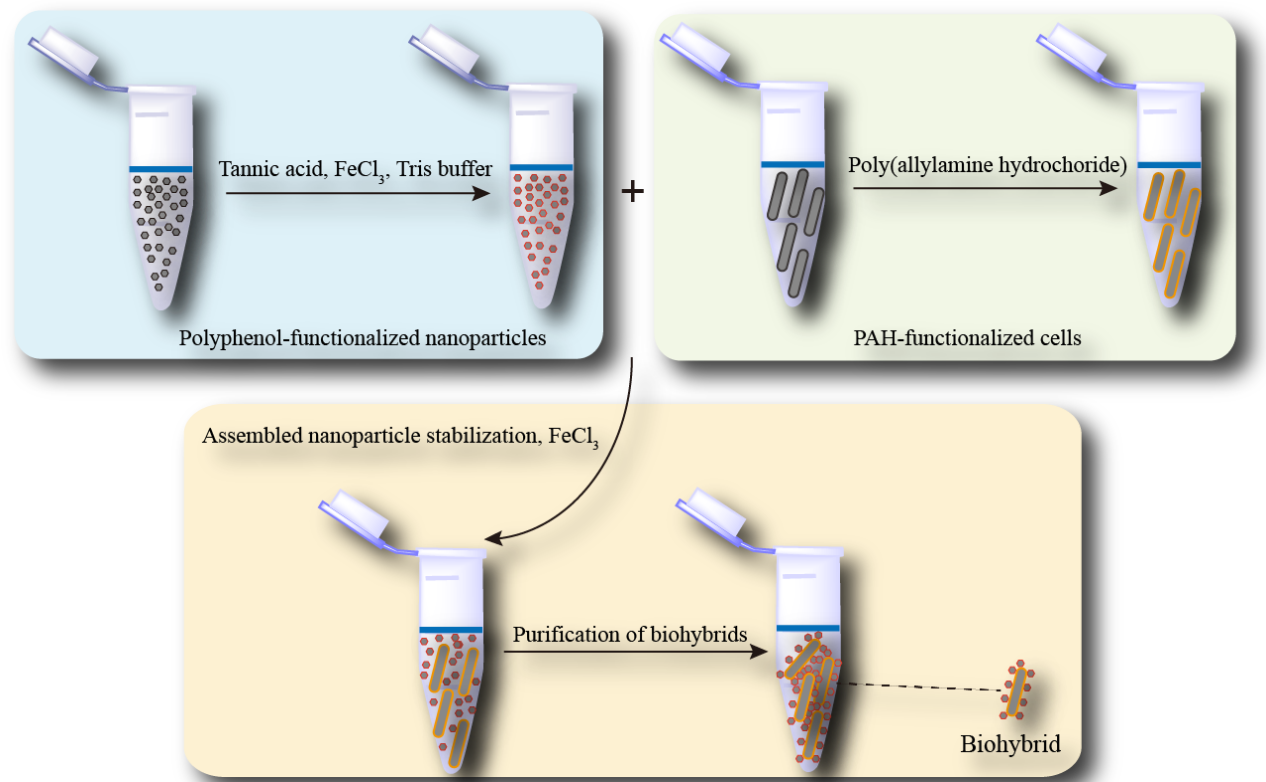
**

**Supplementary Figure. 1 The process of polyphenol-functionalized nanoparticles and PAH-functionalized cells for biohybrid** ((Guo 2018; Sakimoto K K 2016; Wei et al. 2018)).


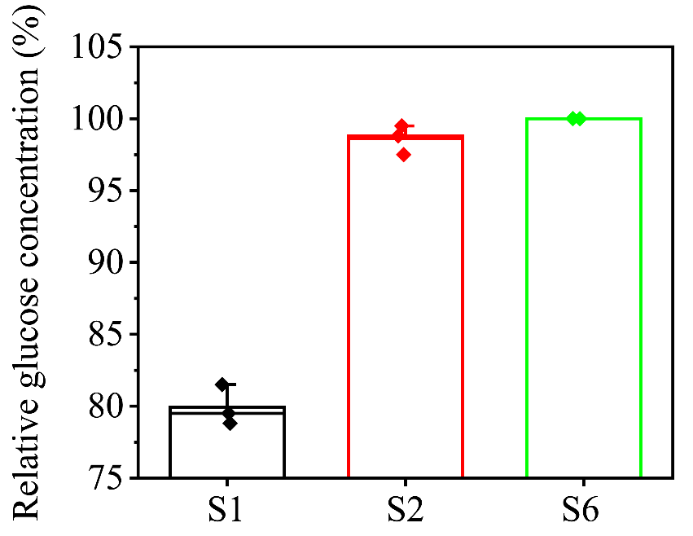


**Supplementary Figure. 2 Comparisons of glucose concentration in different strains.** The starch consumption time has been calculated in different strains. n=3. Error bars, mean ± s.d.


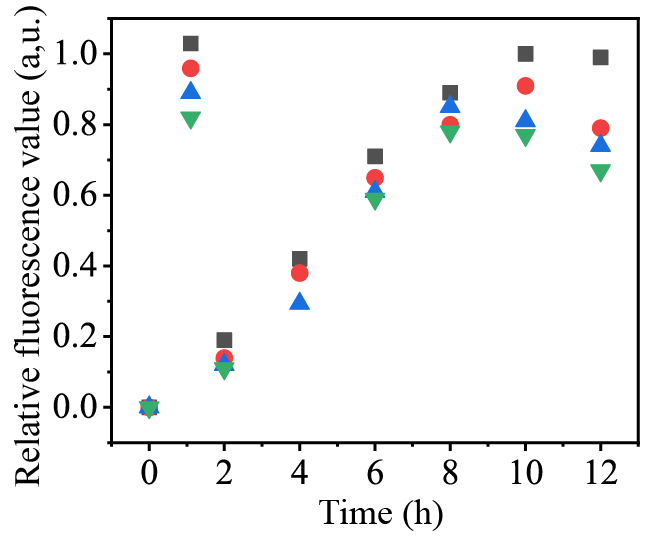


**Supplementary Figure. 3 Comparing the degradation tag for ON and OFF conditions.** Dark color is the LAA tag group for EL222 protein, blue color is the DAS tag for EL222, green color is the GSN tag for EL222, red color is the control group. Dark condition for 0-6 h, light condition for 6-12 h, all values were normalized to the maximum fluorescence value.


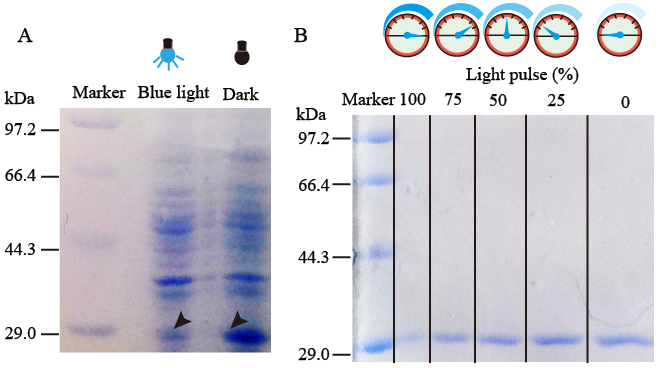


**Supplementary Figure. 4 Comparing the SDS gels of mKate expression under different conditions. (A)** The SDS gels of mKate protein under blue light and dark conditions. **(B)** The SDS gels of purified mKate protein under different blue light pulse.


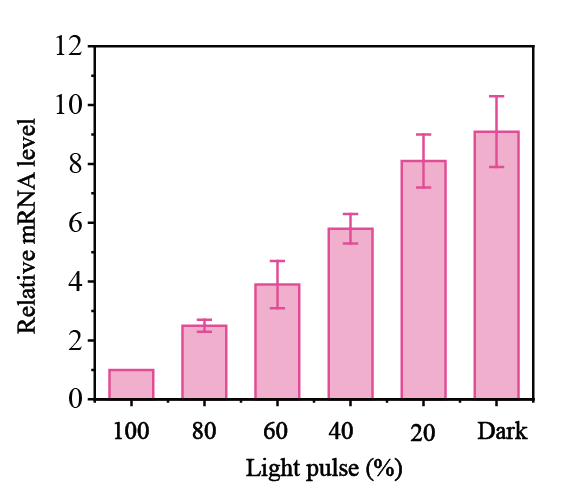


**Supplementary Figure. 5 Comparing the mRNA level of mKate under different blue light pulse.** Different light intensity was used to regulate the expression of mKate, and exhibited the mRNA level in different light pulse. n=2. Error bars, mean ± s.d.

**
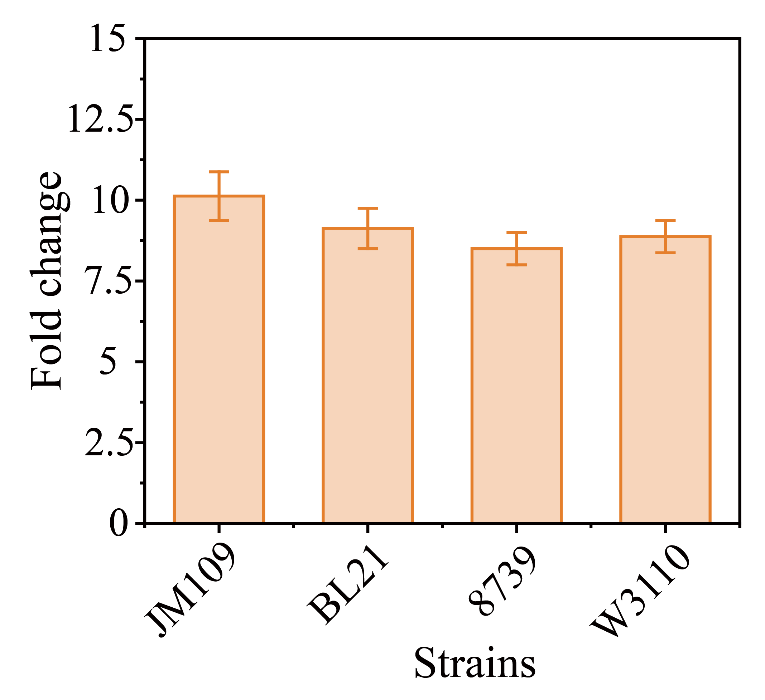
**

**Supplementary Figure. 6 BLRS system used in different *E. coli***. *E. coli* JM109, BL21, 8739, W3110 used for BLRS system. N=3. Error bars, mean ± s.d.

**
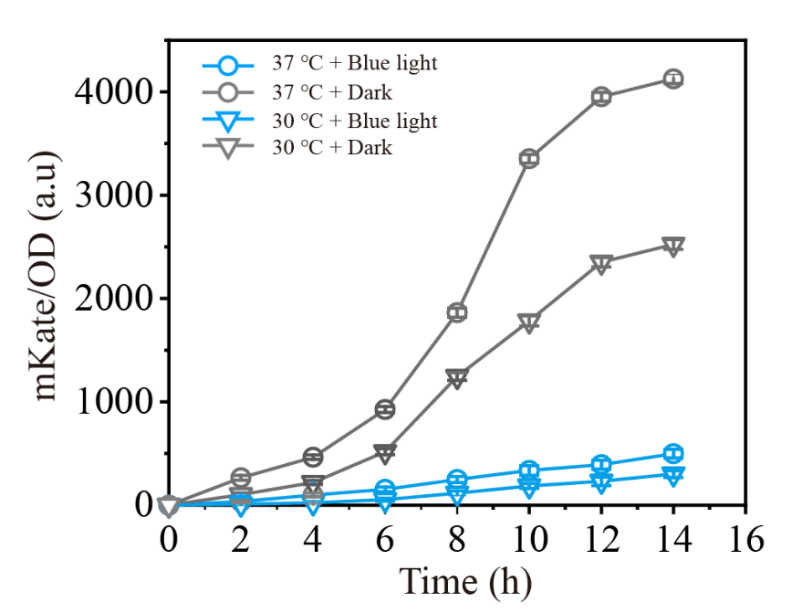
**

**Supplementary Figure. 6 BLRS system used in different temperature**. *E. coli* JM109 with BLRS system used in 37 °C and 30 °C. N=2. Error bars, mean ± s.d.


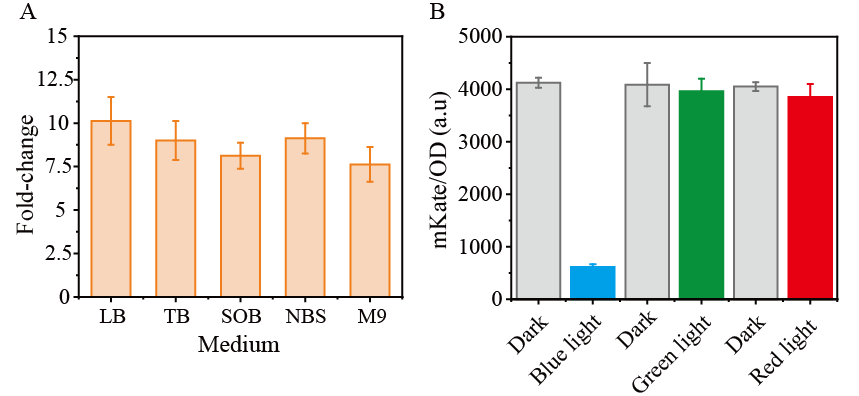


**Supplementary Figure. 7 BLRS system used in different medium and light sources**. *E. coli* JM109 with BLRS system used in different mediums and light sources. N=2. Error bars, mean ± s.d.

**
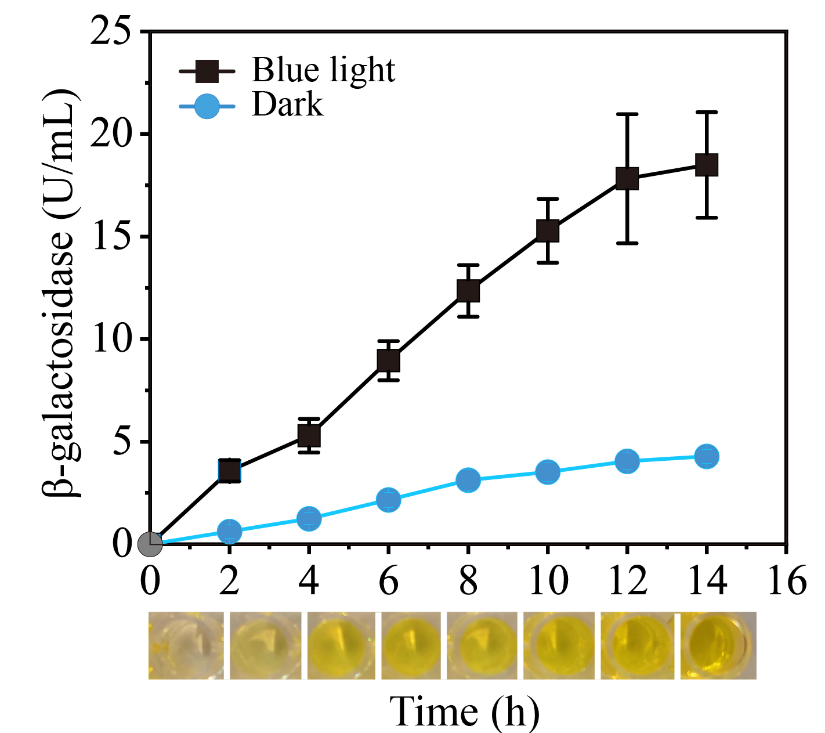
**

**Supplementary Figure. 8 BLRS system used for controlling galactosidase**. *E. coli* JM109 with BLRS system used for controlling galactosidase in the dark and blue light conditions. N=2. Error bars, mean ± s.d.

**
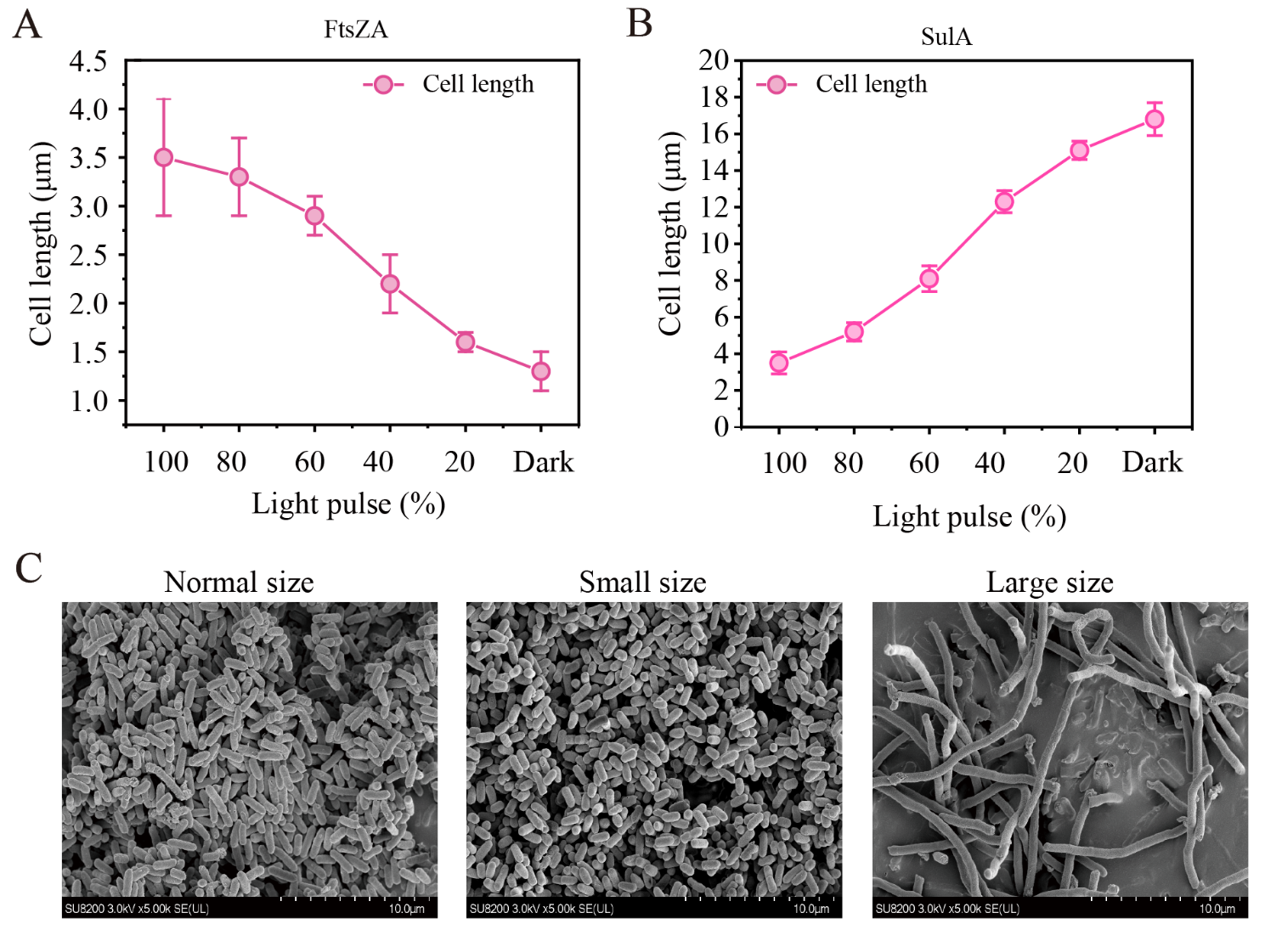
**

**Supplementary Figure. 9 BLRS system used for controlling cell division**. (A) *E. coli* JM109 with BLRS system used for controlling cell division protein FtsZA with different light pulse. (B) *E. coli* JM109 with BLRS system used for controlling cell division inhibitor protein SulA with different light pulse. N=2. Error bars, mean ± s.d.

**
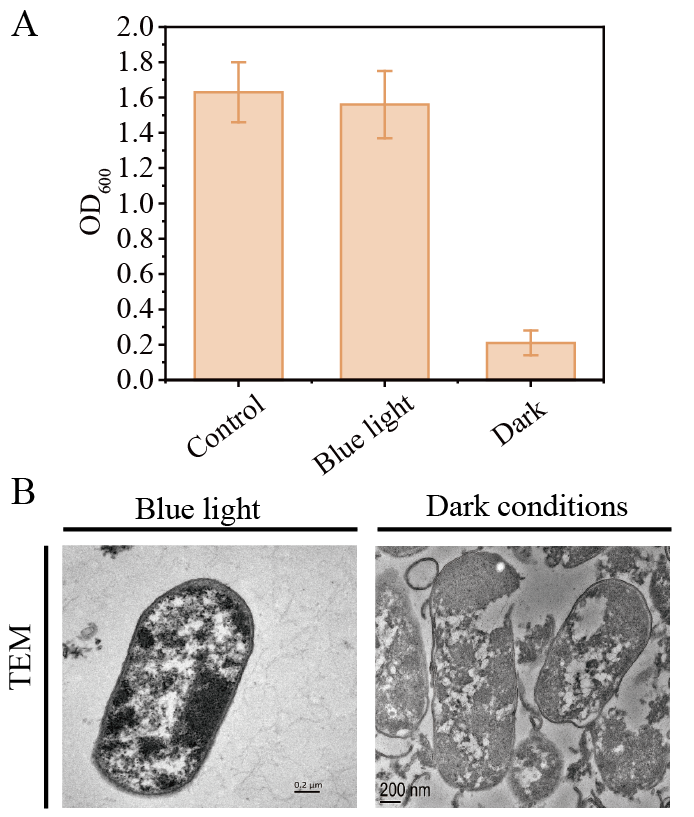
**

**Supplementary Figure. 10 BLRS system used for controlling cell lysis**. (A) *E. coli* JM109 with BLRS system used for controlling cell lysis gene X174E with different light pulse. (B) TEM analysis for cell lysis or normal under the dark condition or blue light.

**
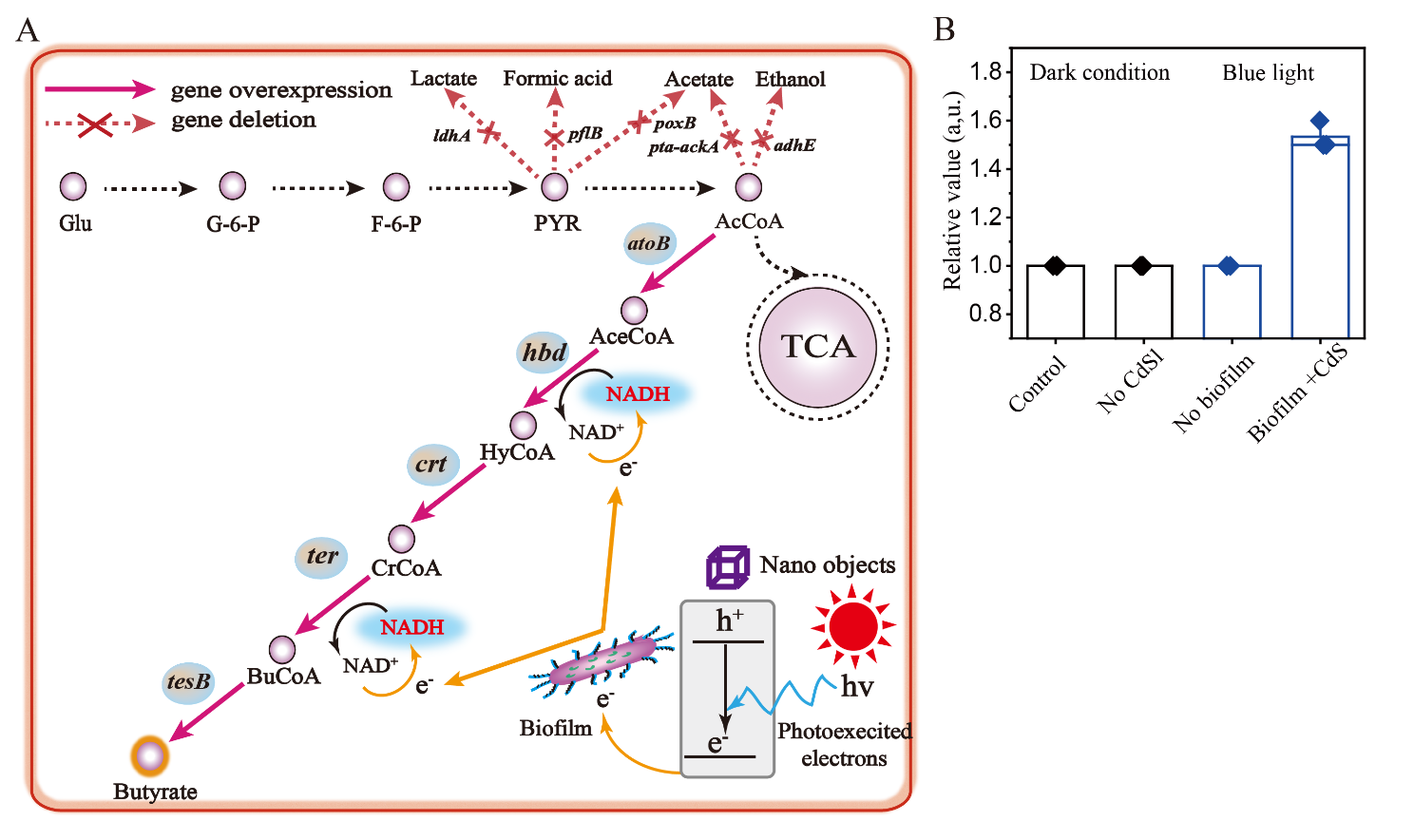
**

**Supplementary Figure. 11 The CdS-biohybrid system used for butyrate production.** (A) Butyrate synthesis pathway in *E. coli*. (B) Relative NADH value with different conditions. N=3. Error bars, mean ± s.d.

**Supplementary Tables**

**Table 1. Amino acid sequences in this study**

| Genes | Sequences |
| --- | --- |
| *el222* | MGADDTRVEVQPPAQWVLDLIEASPIASVVSDPRLADNPLIAINQAFTDLTGYSEEECVGRNCRFLAGSGTEPWLTDKIRQGVREHKPVLVEILNYKKDGT |
| *csgA* | MKLLKVAAIAAIVFSGSALAGVVPQYGGGGNHGGGGNNSGPNSELNIYQYGGGNSALALQTDARNSDLTITQHGGGNGADVGQGSDDSSIDLTQRGFGNSATLDQWNGKNSEMTVKQFGGGNGAAVDQTASNSSVNVTQVGFGNNATAHQY |
| *spy-tag* | LFGGFICIDHNDVR |
| *mKate* | MSELIKENMHMKLYMEGTVNNHHFKCTSEGEGKPYEGTQTMRIKAVEGGPLPFAFDILATSFMYGSKTFINHTQGIPDFFKQSFPEGFTWERVTTYEDGGVLTATQDTSLQDGCLIYNVKIRGVNFPSNGPVMQKKTLGWEASTETLYPADGGLEGRADMALKLVGGGHLICNLKTTYRSKKPAKNLKMPGVYYVDRRLERIKEADKETYVEQHEVAVARYCDLPSKLGHR |
| *gfp* | MVSKGEELFTGVVPILVELDGDVNGHKFSVSGEGEGDATYGKLTLKFICTTGKLPVPWPTLVTTLTYGVQCFSRYPDHMKQHDFFKSAMPEGYVQERTIFFKDDGNYKTRAEVKFEGDTLVNRIELKGIDFKEDGNILGHKLEYNYNSHNVYIMADKQKNGIKVNFKIRHNIEDGSVQLADHYQQNTPIGDGPVLLPDNHYLSTQSALSKDPNEKRDHMVLLEFVTAAGITLGMDELYK |

**Table 2. Elements sequences in this study**

| Elements | Sequences |
| --- | --- |
| P_j_ | TTGACAGGTAGCCTTTAGTCCATGTATAAT |
| S60 | TTCTGTCATATTCCCTATTAGCTGCTATACTTTAAAG |
| fiC | TGCTCTCCCGGCGTAACCCGGATTTGCCGCTTATACTTGTGGC |
| BolA | TAAGCTGCAATGGAAACGGTAAAAGCGGCTAGTATTTAAAGGG |
| J23119 | ttgacagctagctcagtcctaggtataat |
| J23105 | TTTACGGCTAGCTCAGTCCTAGGTACTATGCTAGC |
| J23114 | TTTATGGCTAGCTCAGTCCTAGGTACAATGCTAGC |
| Tac | TTGACAATTAATCATCcGGCTCGTATAATG |
| rrnBT1 | caaataaaacgaaaggctcagtcgaaagactgggcctttcgtttta |
| T5 | TCATAAAAAATTTATTTGCTTTGTGAGCGGATAACAATTATAATA |
| T7 terminator | CTAGCATAACCCCTTGGGGCCTCTAAACGGGTCTTGAGGGGTTTTTTG |
| RBS34 | AAAGAGGAGAAA |
| 20RBS | tagcaggaggaa |
| LAA | GCTGCTAACGATGAAAATTACGCACTGGCAGCT |
| DAS | GCTGCTAACGATGAAAACTATTCTGAGTCCGAATCCGAGAATTACGCAGATGCATCC |
| GSN | GCTGCTAACGATGAAAACTATGGCTCCAATTACGCAGATGCATCC |

**Table 3. Plasmids used in this study**

| Plasmid | Genotype | Source |
| --- | --- | --- |
| pETac | pCole ori, Kan^R^, rrB terminator, lac operator, P_Tac_ | Lab stock |
| pTet | p15A ori, Cm^R^, rrB terminator, ptet operator, P_Tet_ | Lab stock |
| pSC101 | pSC101 ori, Spe^R^, T7 terminator, P_J23119_ | Lab stock |
| pTargetF | pMB1 ori, Spe^R^, rrB terminator, P_J23119_ | Lab stock |
| pTrcHisA | pBR322 ori, Amp^R^, 6*His, rrB terminator, P_trc_ | This study |
| pEM-CsgA | pCole ori, Amp^R^, rrnB terminator, lac operator, P_T5,_ CsgA | This study |
| pEM-CsgB | pCole ori, Amp^R^, rrnB terminator, lac operator, P_T5,_ CsgB | This study |
| pEM-CsgC | pCole ori, Amp^R^, rrnB terminator, lac operator, P_T5,_ CsgC | This study |
| pEM-CsgD | pCole ori, Amp^R^, rrnB terminator, lac operator, P_T5,_ CsgD | This study |
| pEM-CsgE | pCole ori, Amp^R^, rrnB terminator, lac operator, P_T5,_ CsgE | This study |
| pEM-CsgA-Spy | pCole ori, Amp^R^, rrnB terminator, lac operator, P_T5,_ CsgA-Spy | This study |
| pEM-CsgA-kan | pCole ori, Kan^R^, rrnB terminator, lac operator, P_T5,_ CsgA | This study |
| pETac-GFP-Catcher | pCole ori, Kan^R^, rrnB terminator, lac operator, P_Trc,_ GFP-Catcher | This study |
| pETac-SBA-Catcher | pCole ori, Kan^R^, rrnB terminator, lac operator, P_Trc,_ SBA-Catcher | This study |
| ps60-mKate | pMB1 ori, Spe^R^, rrnB terminator, Ps60, RBS34, mKate | This study |
| pfic-mKate | pMB1 ori, Spe^R^, rrnB terminator, Pfic, RBS34, mKate | This study |
| pbolA- mKate | pMB1 ori, Spe^R^, rrnB terminator, PbolA, RBS34, mKate | This study |
| pETac-SBA | pCole ori, Kan^R^, rrnB terminator, lac operator, P_Trc,_ SBA | This study |
| Pj-mKate | pcole ori, Spe^R^, rrnB terminator, Pj, RBS34, mKate | This study |
| ptac-EL222 | pSC101 ori, Cm^R^, rrnB terminator, P_tac,_ RBS34, EL222 | This study |
| pAsAC | CA from *Synechocystis* 6803 cloned in PCK and MDH from *A. succinogenes* cloned in pTrcHisA | **(Hu et al. 2018)** |
| pfic-AsAc | Pfic replaced the Ptac promoter in pAsAC | This study |
| ptac-EL222-LAA | pSC101 ori, Cm^R^, rrnB terminator, P_tac,_ RBS34, EL222, LAA | This study |
| ptac-EL222-GSN | pSC101 ori, Cm^R^, rrnB terminator, P_tac,_ RBS34, EL222, GSN | This study |
| ptac-EL222-DAS | pSC101 ori, Cm^R^, rrnB terminator, P_tac,_ RBS34, EL222, DAS | This study |
| Pcole-atcht | *atoB, tesB, ter, hbd, crt* were cloned into pETac | **(Guo et al. 2020)** |
| pJ01-BGA | pJ01 containing *aroG^fbr^*, *tktA* and *aroB^opt^* | **(Gao et al. 2019)** |
| pETac-SBA-SC | pCole ori, Kan^R^, rrnB terminator, lac operator, P_Trc,_ SBA, P_Trc,_ SBA-Catcher | This study |
| pJ01-BGA-CS | pJ01-BGA adding Pt5, CsgA-Spy | This study |

**Table 4. Strains used in this study.**

| Strain | *E. coli* genotype/plasmid | Source |
| --- | --- | --- |
| *Synechocystis* 6803 | *Wide type* | Wild type |
| *A. succinogenes* | *Wide type* | Wild type |
| M1 | FH0210+pAsAc | **(Hu et al. 2021)** |
| S4 | *E. coli* FMME0008 | Lab storage |
| S5 | S4 carrying pJ01-BGA | **(Gao et al. 2019)** |
| *E. coli* JM109 | *E. coli* JM109 | Lab storage |
| *E. coli* MG1655 | *E. coli* MG1655 | Lab storage |
| S1 | *E. coli* S4 carrying pETac-SBA, pJ01-BGA | This study |
| S2 | *E. coli* S4 carrying pETac-SBA | This study |
| C1 | *E. coli* MG1655 carrying pEM-CsgA | This study |
| C2 | *E. coli* MG1655 carrying pEM-CsgB | This study |
| C3 | *E. coli* MG1655 carrying pEM-CsgC | This study |
| C4 | *E. coli* MG1655 carrying pEM-CsgD | This study |
| C5 | *E. coli* MG1655 carrying pEM-CsgE | This study |
| C6 | *E. coli* MG1655 carrying pEM-CsgA-Spy | This study |
| CSC1 | *E. coli* MG1655 carrying pEM-CsgA-Spy, pETac-GFP-Catcher | This study |
| S60 | *E. coli* JM109 carrying ps60-mKate | This study |
| Fic | *E. coli* JM109 carrying pfic-mKate | This study |
| BolA | *E. coli* JM109 carrying pbolA-mKate | This study |
| M6 | *E. coli* FH0210 carrying pEM-CsgA-kan | This study |
| S5 | *E. coli* S4 carrying pJ01-BGA-CS, pETac-SBA-SC | This study |
| EM1 | *E. coli* JM109 carrying ptac-EL222, Pj-mKate | This study |
| EM2 | *E. coli* JM109 carrying ptac-EL222-LAA, Pj-mKate | This study |
| EM3 | *E. coli* JM109 carrying ptac-EL222-GSN, Pj-mKate | This study |
| EM4 | *E. coli* JM109 carrying ptac-EL222-DAS, Pj-mKate | This study |
| S6 | *E. coli* S4 carrying pJ01-BGA, pETac-SBA-SC, ptac-EL222-Pj- CS | This study |

**Table 5. Primers used in this study.**

| Primers | Sequences | Source |
| --- | --- | --- |
| YZ-tet-F | CCTTCGATTCCGACCTCAT | This study |
| KZ-tac-A | GGATGATTAATTGTCAAATGATGTCTAGATT | This study |
| KZ-tac-S | GGCTCGTATAATGGAATTCGAG | This study |
| YZ-tet-R | GTTCACCGACAAACAACAGATA | This study |
| KZ-RBS34-EL222-A2-1 | GCCTGGAGATCCTTACTCGAGTTAGATTCCGGCTTCGACGG | This study |
| KZ-RBS34-EL222-S2-1 | TATAATGGAATTCGAGCTCCGTCGACAAAGAGGAGAAAACGCG  TATGGGGGCAGACGACACACGCGTTGA | This study |
| pETac-S | GCATGCAAGGAGATGGCG | This study |
| pETac-A | ATCCGGATATAGTTCCTCCTTTCA | This study |
| KZ-GFP-S(RH) | TTCACACAGGAAACAGAATTCATGGTGAGCAAGGGCGAGG | This study |
| KZ-GFP-A(RH) | CTTGTACAGCTCGTCCATGC | This study |
| KZ-spy-catcher-S1 | GCATGGACGAGCTGTACAAGGTCGACGGGAGTGGTGGCAG  CGGAGATAGTGCTACCCATATTAAA | This study |
| KZ-spy-catcher-A1 | CTCGAGTGCGGCCGCAAGCTTGTCGACTTAGCCATTTACA  GTAACCTGAC | This study |
| KZ-CsgA-S | CACCATCACCATCACGGATCCATGAAACTTTTAAAAGTAGC  AGCAATTG | This study |
| KZ-CsgA-A | TCGACCCGGGGTACCGAGCTCTTAGTACTGATGAGCGGTCG  CG | This study |

**References:**

Gao C, Hou JH, Xu P, Guo L, Chen XL, Hu GP, Ye C, Edwards H, Chen J, Chen W and others. 2019. Programmable biomolecular switches for rewiring flux in *Escherichia coli*. Nat Commun 10(1):1-12.

Guo JL, *et al.* 2018. Light-driven fine chemical production in yeast biohybrids. Science 25(10):158-169.

Guo L, Diao W, Gao C, Hu G, Ding Q, Ye C, Chen X, Liu J, Liu L. 2020. Engineering *Escherichia coli* lifespan for enhancing chemical production. Nat Catal 3(3):307-318.

Hu GP, Li ZH, Ma DL, Ye C, Zhang LP, Gao C, Liu LM, Chen XL. 2021. Light-driven CO_2_ sequestration in *Escherichia coli* to achieve theoretical yield of chemicals. Nat Catal:<https://doi.org/10.1038/s41929-021-00606-0>.

Hu GP, Zhou J, Chen XL, Qian YY, Gao C, Guo L, Xu P, Chen W, Chen J, Li Y and others. 2018. Engineering synergetic CO_2_-fixing pathways for malate production. Metab Eng 47:496-504.

Sakimoto K K WAB, Yang P. 2016. Self-photosensitization of nonphotosynthetic bacteria for solar-to-chemical production. Science 20(15-20):74-77.

Wei W, Sun P, Li Z, Song K, Su W, Wang B, Liu Y, Zhao J. 2018. A surface-display biohybrid approach to light-driven hydrogen production in air. Sci Adv 4(2):263-272.
